# Circulating microparticles in acute diabetic Charcot foot exhibit a high content of inflammatory cytokines, and support monocyte-to-osteoclast cell induction

**DOI:** 10.1038/s41598-017-16365-7

**Published:** 2017-11-27

**Authors:** Jennifer Pasquier, Binitha Thomas, Jessica Hoarau-Véchot, Tala Odeh, Amal Robay, Omar Chidiac, Soha R. Dargham, Rebal Turjoman, Anna Halama, Khalid Fakhro, Robert Menzies, Amin Jayyousi, Mahmoud Zirie, Jassim Al Suwaidi, Arash Rafii, Rayaz A. Malik, Talal Talal, Charbel Abi Khalil

**Affiliations:** 1 Stem Cell and Microenvironment Laboratory, Weill Cornell Medicine-Qatar, Doha, Qatar; 2 Department of Genetic Medicine, Weill Cornell Medicine, New York, USA; 3 Department of Genetic Medicine, Weill Cornell Medicine-Qatar, Doha, Qatar; 4 Infectious Disease Epidemiology Group, Weill Cornell Medicine-Qatar, Doha, Qatar; 5 Department of Physiology and Biophysics, Weill Cornell Medicine-Qatar, Doha, Qatar; 6 Sidra Medical and Research center, Doha, Qatar; 7 Department of Podiatry, Hamad Medical Corporation, Doha, Qatar; 8 Department of Diabetes and Endocrinology, Hamad Medical Corporation, Doha, Qatar; 9 Heart Hospital, Hamad Medical Corporation, Doha, Qatar; 10 Department of Medicine, Weill Cornell Medicine-Qatar, Doha, Qatar; 11 Department of Medicine, Weill Cornell Medicine, New York, USA

## Abstract

Circulating microparticles (MPs) are major mediators in cardiovascular complications of type 2 diabetes (T2D); however, their contribution to Charcot foot (CF) disease is not known. Here, we purified and assessed the origin, concentration and content of circulating MPs from 33 individuals: 11 with T2D and acute CF, 11 T2D patients with equivalent neuropathy and 11 non-diabetic controls. First, we demonstrated that there were no differences in the distribution of MPs of endothelial, platelet origin among the 3 groups. However, MPs from leukocytes and monocytes origin were increased in CF patients. Moreover, we demonstrated that monocytes-derived MPs originated more frequently from intermediate and non-classical monocytes in CF patients. Five cytokines (G-CSF, GM-CSF, IL-1-ra, IL-2 and IL-16) were significantly increased in MPs from acute CF patients. Applying ingenuity pathways analysis, we found that those cytokines interacted well and induced the activation of pathways that are involved in osteoclast formation. Further, we treated THP-1 monocytes and monocytes sorted from healthy patients with CF-derived MPs during their differentiation into osteoclasts, which increased their differentiation into multinucleated osteoclast-like cells. Altogether, our study suggests that circulating MPs in CF disease have a high content of inflammatory cytokines and could increase osteoclast differentiation *in vitro*.

## Introduction

Charcot foot (CF) disease is a rare (0.08–7.5%) but extremely debilitating complication in patients with diabetes^1^, which can result in amputation and increased mortality^2^. Indeed the life expectancy of patients with CF is reduced by ~14.4 years^3^. The major risk factor for CF disease is severe peripheral neuropathy leading to sensory loss and failed vasoregulation^4^. There is now an established sequence of events driving uncontrolled and inappropriate inflammation via the release of pro-inflammatory cytokines such as TNF-α, IL-1β, and IL-6, which activate nuclear factor-κB (NF-κB); this in turn induces osteoclast precursor cells to differentiate into mature osteoclasts leading to bone resorption and deformation^5–7^. In a study of monocytes from patients with acute CF, there was increased production of pro-inflammatory cytokines, reduced secretion of anti-inflammatory cytokines, increased expression of surface co-stimulatory molecules and increased resistance to serum withdrawal-induced apoptosis^8^.

It is now becoming increasingly recognized that cells release extra-cellular vesicles into their surrounding environment. This heterogeneous population, different from apoptotic bodies, are referred to as microparticles (MPs) and display a size ranging from 100 nm to a few micrometers in diameter^9^. These MPs are known to express a large number of surface markers, which interact as intercellular communication messengers between the parent cell and different targeted cells. In healthy individuals, MPs in the plasma are derived from leukocytes, erythrocytes, platelets and endothelial cells (ECs)^10,11^. Endothelial microparticles (EMPs) generally circulate at a relatively low level and reflect normal endothelial cell turnover^12–14^. Elevated circulating levels of EMPs have been identified in a number of cardiovascular disease (CVD) pathologies including renal failure, acute coronary syndrome, stroke, venous thromboembolic disease, metabolic syndrome and severe hypertension^10,15,16^.

Since MPs are also known to be deregulated in patients with T2D, we hypothesized that they may play a role in CF. Here; we demonstrated that MPs isolated from the plasma of patients with CF differ in their origin as compared to healthy controls or patients with diabetic neuropathy (DN). While MPs from an endothelial origin were the same, leukocytes - and more precisely monocytes- derived MPs were higher in CF patients. Moreover, they contain more G-CSF, CM-CSF, IL-16, IL-2 and IL-RA cytokines. Finally, treating monocytes THP-1 or monocytes sorted from healthy patients) with CF-derived MPs induced the expression of the downstream target of these cytokines and support osteoclastic differentiation *in vitro*.

## Research Design and Methods

### Subjects

11 T2D patients with acute CF, matched for age, gender and BMI with 11 T2D patients with neuropathy but no CF and 11 non-diabetic controls were studied. T2D patients were also matched for HbA_1c_. CF patients were recruited from the podiatry department at Hamad Medical Corporation (HMC), Doha-Qatar. Patients with DN were recruited from the department of endocrinology and diabetes in HMC. Healthy controls were recruited from the waiting area in the outpatient department.

Acute Charcot foot was diagnosed according to the American Diabetes Association and the American Podiatric Medical Association task force^17^. Patients had to have a red swollen foot with increased local temperature of more than 2 °C compared to the contralateral foot with X-Ray evidence of acute Charcot foot. Foot temperature was measured using FLUKE Ti32 thermal imager (Fluke Corporation - USA). Among the 11 patients with acute CF, 4 had dislocations or subluxations, 2 had fractures and 5 had erosions and degenerative changes. DN was diagnosed based on the vibration perception threshold (Neurothesiometer NU-1, Horwell- UK) on the great toe being >25 V^18^.

Venipuncture was performed from the forearm veins and blood was transported immediately from HMC to the research lab.

All participants provided written informed consent approved by Weill Cornell Medicine-Qatar and Hamad Medical Corporation IRBs (13-00031 and 14-14054, respectively). The study was conducted in accordance with the 1964 Declaration of Helsinki.

### Microparticle sampling

#### Separation of plasma from whole blood

10 ml of blood was collected in 2 × 5 ml heparin vacutainer (green cap). Centrifugation was performed at 800 rpm for 10 min at 4 °C. After centrifugation, the supernatant (plasma) was removed using clean pipette technique and placed in a labeled eppendorf tube.

#### MPs purification

MPs isolation was performed using ExoQuick^TM^-LP (System Biosciences, Mountain View, CA) according to the manufacturer recommendations^19,20^. ExoQuick-LP contains pre-clearing reagents to deplete efficiently lipoprotein particles from plasma before ExoQuick precipitation. Briefly, plasma was centrifuged at 4 °C and 3,000 g for 15 min to remove cells and cell debris. Aliquots of 250 µL were mixed with 63 µL of ExoQuick^TM^-LP and incubated at 4 °C overnight. The mixture was centrifuged at 1,500 g for 30 min and the MP pellets were collected. The final pellet containing purified MPs was either re-suspended in media for treatment of cell cultures or lysed for protein extraction or labeled for cytometry analysis or microscopy imaging. The protein concentrations of MPs were measured by Bradford assay (Biorad).

### THP-1 cell culture

The Human THP-1 monocyte cell line was purchased from ATCC (Manassas, VA, USA) and cultured in RPMI 1640 (Sigma Life Science) supplemented with 10% fetal bovine serum (Sigma Life Science), 1% Penicillin-Streptomycin-Amphotericyn B solution (Sigma) and 0.0004% β-mercaptoethanol. Cultured cells were incubated at 37 °C under a water-saturated 95% air-5% CO2 atmosphere and the media was replaced every 3 days.

### Osteoclast differentiation

Adhesion of THP-1 was first induced by seeding 5 × 10^4^ cells in the well of a 48 well-Plate (Falcon, Becton Dickinson) and incubated with 100 mM PMA (phorbol myristate acetate) for 48 hours. Then, the medium was changed and cells were incubated with MCSF (25 ng/mL) and RANKL (50 ng/mL) for 14 days. Revelation was performed using TRAP assay (leukocyte acid phosphatase, 387 A Sigma Aldrich) as per the manufacturer recommendation. Acid Phosphatase activity appears as purplish to dark red granules in the cytoplasm. TRAcP-positive cells with more than three nuclei were identified as multinucleated osteoclast-like cells. The number of newly generated osteoclasts was assessed using light microscopy (EVOS^®^FL Imaging System).

### Flow cytometry

To evaluate their endothelial origins, MPs extracted from plasma were stained with an anti-human PECAM (CD31-FITC, clone WM59, optimized condition) joined to fluorescein and an anti-human E-selectin (CD62E-PE, clone 68-5H11; optimized condition) joined to phycoerythrin. Anti-human CD42b (CD42b-APC, clone HIP1; optimized condition) joined to phthalocyanine was placed (45 min, 4 °C) into each specimen to exclude MPs of platelet origin. Singly and doubly positive CD42b^−^CD31+CD62+MPs were assessed by simultaneously incubating the MPs with all 3 antibodies as described above. All MP measurements were carried out twice to ensure repeatability. CD42b^−^CD31+ and CD42b^−^CD62+MPs levels were optimized using control antibodies. Anti-human CD45-PECy5 (leukocyte marker, clone HI30; optimized condition) were utilized to assess contamination by white blood cell derived MPs. MPs < 1.5 μm were identified by size and density by forward and sideways light scatter plots utilizing polystyrene microspheres (0.2 to 10 μm) to calibrate size, and assessed by 2- or 3- color fluorescence histogram plots as CD42b^−^CD31+ or CD42b^−^CD62+MPs (Supplementary Figure 1).

To evaluate the origin of MPs from peripheral blood mononuclear cell (PBMCs), MPs were stained with CD45-PE (BD biosciences) for 45 minutes on ice. CD45^+^MPs represent the leukocytes MPs. To evaluate the monocytes origin, MPs were then stained with the following antibodies: mouse anti-human IgG2b CD14 –APC, mouse anti-human IgG1 CD16-FITC and mouse anti-human CD142-PE (BD bioscience) and incubated for 45 minutes on ice in the dark. MPs were gated as CD142^+^ and CD14/CD16 were evaluated within the CD142^+^.

Fluorescence (FL) was quantified on a SORP FACSAriaII (BD Biosciences). Data were processed with FACSDiva 6.3 software (BD Biosciences). Doublets were excluded by FSC-W × FSC-H and SSC-W × SSC-H analysis. FITC fluorescence was acquired with 488 nm blue laser and 510/50 nm emission; APC conjugated antibody was acquired with 647 nm red laser and 670/14 nm emission and PE fluorescence was acquired with 535 nm green laser and 582/15 nm emission. The charts display the median of fluorescence intensity (mfi) relative to control. Single stained channels were used for compensation and fluorophore minus one (FMO) controls were used for gating. At least 20,000 events were acquired per sample.

### Monocytes isolation

Monocytes were separated from peripheral blood mononuclear cells (PBMCs). The PBMCs were isolated from 2 × 5 mL of blood using heparin vacutainer (green cap). The blood was then diluted to 1:2 ratio with a phosphate buffer saline (PBS) solution, including rinsed vacutainer. 10 mL of diluted blood was overlayed with 5 mL of Ficoll and spun at 400 × g for 30 minutes at room temperature (brake OFF). The whitish-opaque cell layer in the interphase was removed. The interphase was diluted to 1:3 ratio with PBS and spun at 350 × g for 10 minutes at room temperature (brake ON). The supernatant was discarded and pellet was re-suspended in 25 mL of PBS and spun at 200 × g for 15 minutes at room temperature. The platelet rich supernatant was removed by spinning at 200 × g for 15 minutes until the supernatant was clear. Once clear, the supernatant was removed and re-supended in 25 ml PBS, aliquots were taken to count cells and spun again at 200 × g for 15 min at RT. The supernatant was removed completely and depending on the total cell number, the cells were re-suspended as 10^7^ cells per 100 µl in prepared ice-cold blocking buffer and was put on ice.

#### Flow cytometry and sorting

10% of a FcR blocking reagent (Myltenyi Biotec) was added and incubated for 10- 30 minutes on ice. 100 μl of staining buffer was added per 10^7^ of cells. The PBMCs were then stained with the following antibodies: mouse anti-human IgG2b CD14 –APC and mouse anti-human IgG1 CD16-PE (BD bioscience) and incubated for 30–45 minutes on ice in the dark. Finally, the cells were spun at 200 × g for 15 minutes and the pelleted PBMCs were resuspended in 1 mL of staining buffer for sorting. Fluorescence (FL) was quantified on a SORP FACSAria2 (BD Biosciences). Data were processed with FACSDiva 6.3 software (BD Biosciences). Doublets were excluded by FSC-W × FSC-H and SSC-W × SSC-H analysis (Supplementary Figure 4). PE fluorescent was acquired by 498 nm blue laser and 575/26 nm emission; APC fluorescence was obtained by 650 nm red laser and 660/20 nm emission; Figures display the median of fluorescence intensity (mfi) relative to control. Single stained channels were used for compensation and fluorescence minus one (FMO) controls were used for gating. Purity of the sorting was controlled after each sorting and was > 90%.

### Confocal Microscopy

To visualize MP uptake by THP-1, MPs were stained with Alexa Fluor 594 conjugated wheat germ agglutinin (WGA AF594) and cultured for 24 h at 37 °C. The THP-1 cells treated with MPs were fixed in 3.7% formaldehyde and the slides were mounted in a mounting media SlowFade® Gold Antifade Reagent (Invitrogen). Fluorescence Imaging was performed using a Zeiss confocal Laser Scanning Microscope 710 (Carl Zeiss). Post-acquisition image analysis was performed with Zeiss LSM Image Browser Version 4.2.0.121 (Carl Zeiss).

### Western Blot analysis

Immunostaining was carried out using goat monoclonal antibodies against CD142, IL-1RA (Cell signaling #47769, #3865 S), IL16, GM-CSF, G-CSF, Gro α, CD16 (abcam #ab18673, #ab54429, #ab181053, #ab124898, #ab203883). A secondary anti-rabbit IgG, HRP-linked antibody (Cell Signaling # 7074P2) was used for rabbit antibodies and a secondary anti-mouse IgG, HRP-linked antibody (Cell Signaling #7076 S) was used for mouse antibodies. Blots were developed using SuperSignal West Pico PLUS Chemiluminescent Substrate (#34580 ThemoFisher). Data were collected using the ChemiDoc^TM^ MPs Imaging system (*BioRad)*, and analyzed using Image Lab Software.

### RT-PCR analysis

Total RNA was extracted from cell cultures using Trizol. After genomic DNA removal by DNase digestion (Turbo DNA free kit, Applied Biosystems), total RNA (1 µg) was reverse transcribed with oligodT (Promega) using the Superscript III First-Strand Synthesis SuperMix (Invitrogen). PCR analysis was performed as previously described^20^ with a MasterCycler apparatus (Eppendorf) from 2 µL of cDNA using primers from IDT (Supplementary Table 1).

### Ingenuity pathway analysis

We used Ingenuity Pathway Analysis software (Ingenuity Systems) to annotate and functionally characterize the possible role of the cytokines. Network diagrams were made using Ingenuity software (Qiagen).

### Cytokine and angiogenesis Array

200 μg of protein was loaded on Proteome Profiler™ Human Angiogenesis or Proteome Profiler Human Cytokine Array Kit (R&D Systems®) according to manufacturer’s instructions. Arrays were revealed using HorseRadish Peroxidase (HRP) and SuperSignal West Pico Chemiluminescent Substrate (Thermo-Scientific, Dubai, Emirates). Data were collected using Geliance CCD camera (Perkin Elmer, MA), and extracted using ImageJ software (NIH). Briefly, the pictures of the arrays were inverted and background subtracted. We then defined the area for signal capture for all spots as 110–120 micron diameter, using the same area for every spot. We defined our signal as the median pixel density value. For comparison, the independent arrays values were normalized on their positive control intensity value.

### Statistical analysis

All quantitative data were expressed as mean ± standard error (SD) or stand error of the mean (SEM). Statistical analysis for the cytokine array was performed with Stata 13 (StataCorp LP, Texas). Non-parametric analysis using the Kruskal-Wallis test was performed to compare the levels of each cytokine in the MPs between the three groups. Data trends were visually evaluated for each MP and cytokine. Others Statistical analyses were performed using SigmaPlot 11 (Systat Software Inc., Chicago, IL). A Shapiro-Wilk normality test, with a p = 0.05 rejection value, was used to test normal distribution of data prior to further analysis. All pairwise multiple comparisons were performed by one way ANOVA followed by Holm-Sidak posthoc tests for data with normal distribution or by Kruskal-Wallis analysis of variance on ranks followed by Tukey posthoc tests, in case of failed normality test. Paired comparisons were performed by Student’s t-tests or by Mann-Whitney rank sum tests in case of unequal variance or failed normality test. Statistical significance was accepted for *p < 0.05, **p < 0.01 or ***p < 0.001.

## Results

### Participant characteristics

Our study group comprised 33 individuals (16 women and 17 men). The three groups were defined as follows (i) Control-comprised of non-diabetic controls, (ii) DN- diabetic patients with neuropathy, (iii) CF- diabetic patients with an acute Charcot foot. General characteristics of the study population are shown in Table 1. Age, gender and BMI distribution were similar between the three groups. Diabetes duration and HbA_1c_ did not differ between the DN and CF groups. The number of leukocytes and lymphocytes did not differ between the 3 groups; however, the number of monocytes is increased in CF patients which is consistent with the characteristics of this pathology.

**Table 1:** Characteristics of the participants.

Characteristic	Control n = 11	DN n = 11	CF n = 11	P value
Age, years	57.7 (4, 3)	56.4 (3.6)	56.0 (3.3)	0.94
Gender, F/M	6/5	5/6	5/6	0.89
BMI, kg/m^2^	31.1 (2.1)	30.3 (1.8)	29.6 (2.1)	0.99
HbA1c, %	5.3 (0.2)	7.8 (0.4)	7.3 (0.5)	0.48^#^
Duration, years	N/A	14 (2.9)	17 (2.2)	0.35^#^
Monocytes (%)	6.7 (1.4)	7.2 (1.4)	9.8 (1.8)	0.0014**
Lymphocytes (%)	30.7 (9.0)	35.7 (3.5)	25.3 (8.5)	0.024
Leukocytes (×10^3^)	7.2 (1.7)	9.4 (2.5)	8.3 (2.6)	0.14

Data are presented as mean (SD). DN: Diabetic neuropathy, CF: Charcot foot disease. ^#^For the p value of comparison only between DN and CF groups.

### Characterization of MPs origin and concentration

**Figure 1:**
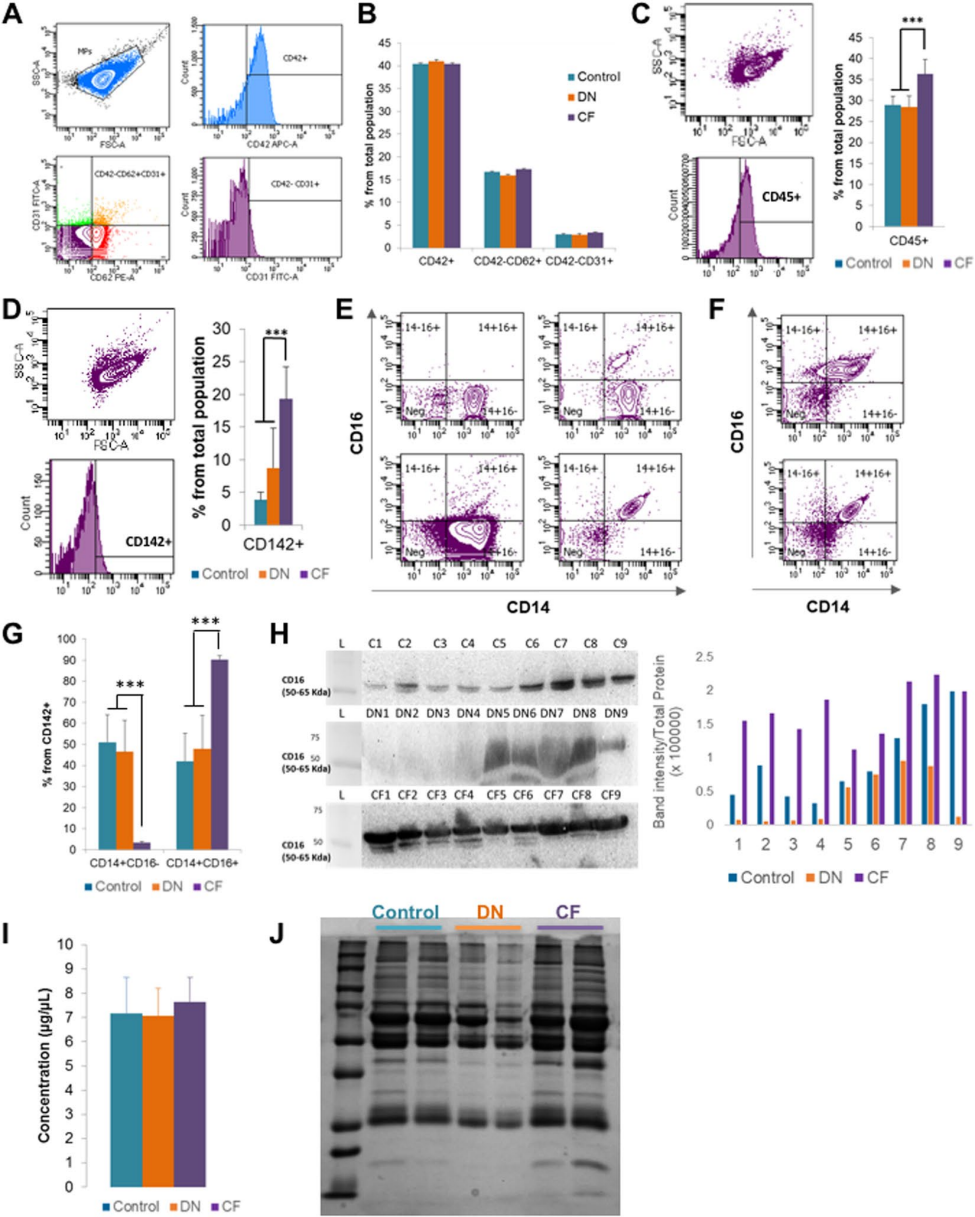
MPs origin characterization. (**A**) Flow cytometry plots of the gating protocol used to identify MPs populations. MPs from healthy controls were stained with CD42-APC, CD62-PE and CD31-FITC. Gate limits were drawn on a control isotype for each antibody. The same strategy was used for the rest of the samples. (**B**) Quantifications of the MP populations in the 3 groups, no statistically significant difference was observed. (**C**) Flow cytometry for CD45 staining on MPs. MPs from healthy controls, DN or CF patients were stained with CD45-PE (The plots represents one of the CF patients). Quantifications of the MP populations in the three groups is shown by the histogram plot on the right. (**D**) Flow cytometry for CD142 staining on MPs. MPs from healthy controls, DN or CF patients were stained with CD142-PE. The plots represent one of the DN patients. Quantifications of the MP populations in the three groups is shown by the histogram plot on the right. (**E**,**F**) Typical flow cytometry charts observed for healthy controls (**E**), DN (**E**) and CF (**F**) patients. MPs were stained with CD142-PE, CD14-APC and CD16-FITC. The plot represents the population staining CD14-CD16 within the CD142^+^ population as defined in D. (**G**) Quantifications of the MP populations based on CD14-CD16 staining within the CD142^+^ population as defined in D. (**H**) CD16 expression in MPs by western blot. The full blot of this cropped blot is presented in Supplementary Figure 5. The total protein measurement done by red Ponceau was used as loading control to normalize the band intensity on the right histogram. (**I**) Protein quantification from the MPs using the Bradford assay in the three groups, no difference in protein concentrations noted. (**J**) Electrophoresis performed on MPs. The distribution of proteins is different in MP contents between the three groups. *p < 0.05, **p < 0.01 or ***p < 0.001.

To evaluate their origins, MPs extracted from plasma were stained with CD31-FITC, CD62E-PE, and CD42b-APC (Fig. 1A and Supplementary Figure 1). MPs from platelets (CD42^+^) did not differ between the three groups, with a proportion of 40.35 ± 1.15%, 40.95 ± 0.51% and 40.67 ± 1.65% for the control, DN and CF groups, respectively, p = 0.95 (Fig. 1B). Similarly, endothelial MPs (CD42^−^/CD31^+^) did not differ between the three groups (p = 0.45).

A higher ratio of CD42b^−^CD62+ to CD42b^−^CD31+ denotes “activated” endothelial MPs and a low ratio denotes “apoptotic” endothelial MPs^15^. However, this ratio was not different in our studied groups (6.05 ± 0.56, 6.5 ± 0.88 and 5.36 ± 0.43 for control, DN and CF group; respectively, p = 0.23). We evaluated the proportion of leukocytes origin MPs using CD45 expression, a known marker of leukocytes (Fig. 1C). MPs from leukocytes are present at a higher amount in CF patients (28.97 ± 2.27%, 28.43 ± 2.62% and 36.3 ± 3.43% for the control; DN and CF groups; respectively; p < 0.001). Since monocytes are of high interest in CF disease, we went further and investigated the monocyte origin of our circulating MPs. CD142+ MPs were higher in CF patients (3.84 ± 1.48%, 8.75 ± 6.04% and 19.28 ± 4.94% for the controls, DN and CF groups; respectively; p < 0.001, Fig. 1D). Many studies have classified human monocytes according to their expression of CD14 and CD16 as follow: classical CD14^++^CD16^−^, intermediate CD14^++^CD16^+^ and non-classical CD14^+^CD16^++^ monocytes^21^. In Fig. 1E, we displayed the four representative charts for CD14/CD16 population (within the CD142^+^ population) that reoccurred while analyzing MPs from the controls and DN groups. MPs were either CD14^++^CD16^-^ or CD14^+^CD16^-^ (left plots) or CD14^+^ with a small part CD16^+^ (upper right plot) or completely CD14^+^CD16^+^ (bottom right plot). In Fig. 1F, we represented the representative charts for CF patients. In CF patients, MPs were CD16^+^. Interestingly, more than half of the patients were having a second population with a lower fluorescence of CD14 (top chart) detaching. Even though, the fluorescence of CD16 is not increasing in this population, we hypothesized that this population could represent MPs coming from non-classical monocytes. When we took a closer look at the ratio within the CD142+population, CF patients displayed a higher proportion of CD14^+^CD16^+^ monocytes as compared to control and DN patients (42.08 ± 13.17%, 47.88 ± 15.95% and 90.31 ± 1.74% for the controls, DN and CF groups respectively; p < 0.001, Fig. 1G). Lastly, we confirmed the presence of CD16 on our circulating MPs using western blot on nine patients in each group (Fig. 1H). Total protein with red Ponceau staining had been used for loading control, the band intensity normalized to the total protein for each lane is displayed on the histogram (right). The results indicate that every CF patient display a high level of CD16.

Finally, there was no significant difference in the full protein concentration between the three groups (p = 0.77; Fig. 1I). Nevertheless, electrophoresis with Coomassie blue staining on two patients from each group demonstrated a difference in protein type (Fig. 1J).

### Characterization of MP content

We first used a Proteome Profiler™ Human Angiogenesis Array Kit to determine the relative levels of 55 human angiogenesis-related proteins (Supplementary Figure 2A). Data trends were evaluated for each cytokine by measuring the pixel density (Supplementary Figure 2B). We couldn’t find any difference in the MPs content expression pattern between the patients from the three different groups (Supplementary Figure 2C). We then analyzed more closely the differences using Stata 13 (Supplementary Table 2). None of the proteins was significantly up or down regulated in the group CF.

**Figure 2:**
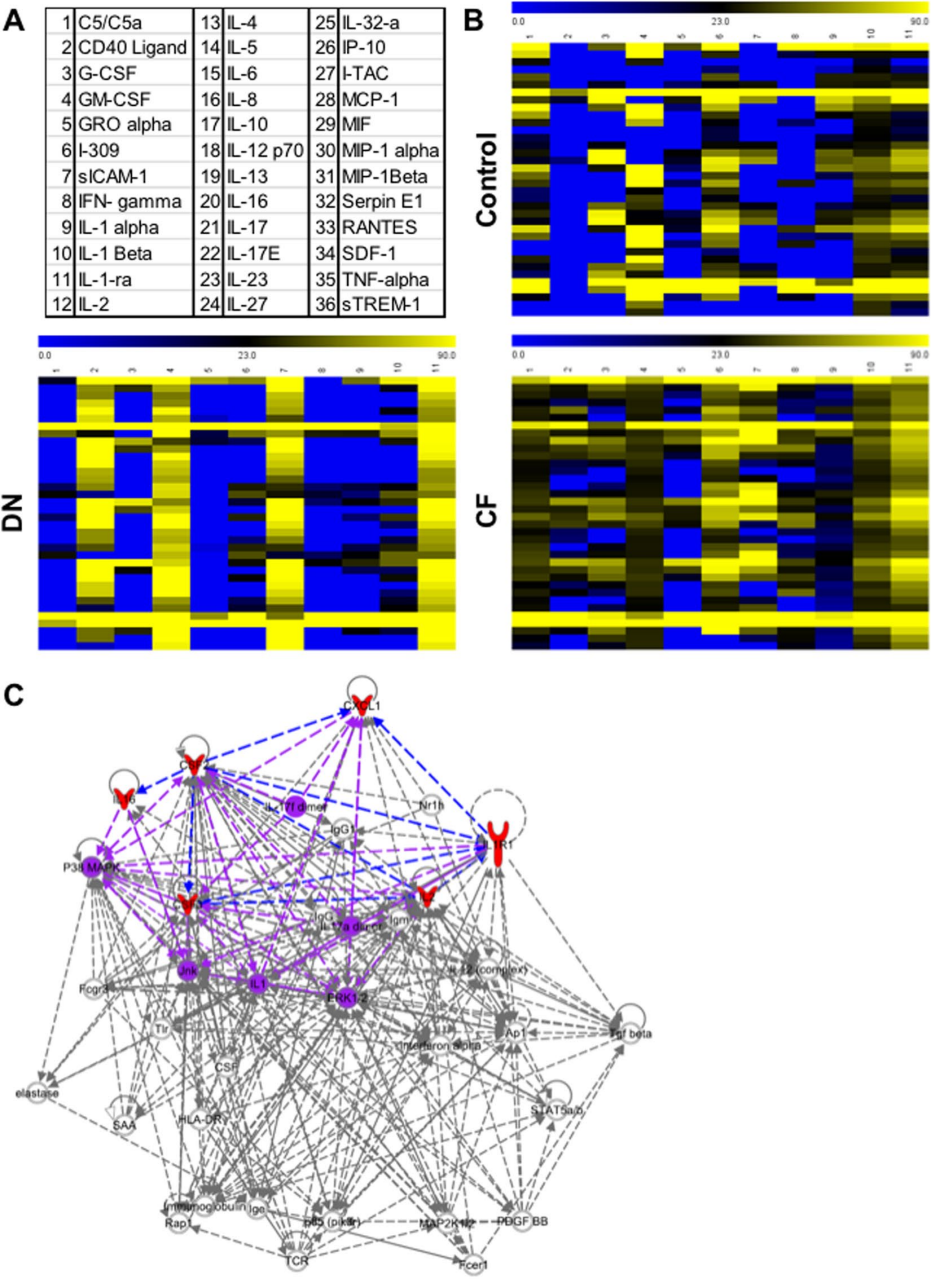
MPs contents characterization. (**A**) List of cytokines tested in the cytokine array. (**B**) Each column in the cluster graphics represents one patient and each line a cytokine. The order of cytokines is similar to the distribution in 2.A and the intensity ranges from blue (less expressed) to yellow (more expressed). MPs extracted from Charcot patients (bottom right graphic) are more complex than the ones from healthy controls (top graphic) or diabetics with neuropathy (bottom left graphic). (**C**) Ingenuity pathway representation. The 6 cytokines that are significantly more expressed in MPs of Charcot patients (red) interact with each other (blue arrows). The interaction between those cytokines is able to activate pathways involved in osteoclast formation (purple).

We then performed Human Cytokine Arrays to simultaneously detect the relative levels of 36 different cytokines, chemokines, and acute phase proteins (Fig. 2A). Using a hierarchical clustering on the samples (Fig. 2B), we demonstrate a difference in MPs content between patients in each group. CF-derived MPs were more different cytokines than the two other groups. Differences in pixel density were analyzed using Stata 13 (Table 2). Five cytokines were significantly increased in CF-derived MPs: G-CSF, GM-CSF, IL-1-ra, IL-2 and IL-16 (p < 0.05) and Gro-α was at the limit of significance (p = 0.054). Using ingenuity pathways analysis, we visualized the interaction of these six cytokines between each other (Fig. 2C, red filled cytokines and blue arrows). Of relevance, the interaction between them induced the activation of pathways (IL-17, IL-1, P38 MAPK, Jnk or ERK1/2) involved in osteoclast formation (purple on Fig. 2C)^22,23^. Using human Cytokine Arrays, we assessed the serum level of these six cytokines and were not able to detect them (Data not shown).

**Table 2:** Cytokines array between the studied groups.

	CF N = 11		DN N = 11		Control N = 11		P-value
	Mean (SD)	Median	Mean (SD)	Median	Mean (SD)	Median	
C5/C5a	133.89 (105.78)	100.27	99.78 (70.33)	85.63	107.57 (94.09)	97.31	0.66
CD40 Ligand	42.79 (18.64)	37.93	33.71 (42.24)	20.55	29.18 (35.55)	26.30	0.11
G-CSF	33.15 (12.07)	33.64	26.92 (49.47)	0.00	6.89 (12.12)	0.00	0.007**
GM-CSF	25.93 (14.91)	22.50	15.14 (28.02)	0.00	5.10 (8.19)	0.00	0.013*
GRO alpha	38.08 (20.17)	33.11	20.13 (30.65)	0.00	16.49 (19.62)	0.00	0.0543*
I-309	26.19 (19.84)	28.58	16.90 (28.96)	0.00	19.09 (13.63)	22.05	0.40
sICAM-1	163.62 (143.36)	144.48	166.38 (71.57)	152.31	159.57 (68.51)	145.58	0.79
IFN- gamma	53.28 (53.10)	37.54	46.49 (30.28)	49.01	63.19 (55.28)	60.46	0.90
IL-1 alpha	61.15 (27.03)	52.90	43.72 (51.11)	18.17	48.46 (49.08)	44.74	0.33
IL-1 Beta	45.61 (16.67)	43.44	29.48 (44.00)	0.00	26.98 (37.86)	18.06	0.09
IL-1-ra	39.48 (15.83)	35.82	29.99 (55.12)	0.00	16.83 (21.63)	13.55	0.025*
IL-2	26.25 (15.73)	22.96	16.61 (30.34)	0.00	5.35 (9.35)	0.00	0.019*
IL-4	19.43 (17.75)	24.55	12.59 (25.56)	0.00	8.69 (10.13)	0.00	0.27
IL-5	18.88 (16.10)	24.13	11.34 (21.89)	0.00	16.94 (14.68)	21.57	0.34
IL-6	31.42 (26.25)	28.17	21.38 (19.79)	17.35	37.98 (33.88)	32.73	0.46
IL-8	39.59 (40.70)	28.64	25.22 (20.76)	24.52	46.66 (43.20)	45.46	0.53
IL-10	63.47 (29.39)	57.86	49.68 (53.94)	22.23	49.72 (51.62)	50.65	0.37
IL-12 p70	44.47 (18.92)	33.99	30.59 (45.02)	0.00	27.87 (38.06)	16.41	0.23
IL-13	56.53 (18.47)	57.10	42.66(58.41)	13.72	31.72 (34.11)	30.62	0.12
IL-16	39.69 (15.78)	39.41	26.40 (42.96)	0.00	12.90 (15.98)	0.00	0.019*
IL-17	21.03 (16.77)	25.23	13.77 (28.14)	0.00	11.12 (14.25)	0.00	0.21
IL-17E	20.09 (15.29)	28.13	18.90 (26.78)	0.00	22.00 (16.76)	25.27	0.70
IL-23	28.45 (18.56)	29.32	23.15 (18.62)	26.65	35.09 (26.57)	34.04	0.52
IL-27	33.59 (27.73)	28.91	24.03 (19.37)	22.30	42.02 (37.84)	29.18	0.45
IL-32-a	72.77 (35.71)	65.86	66.88 (56.64)	49.36	61.86 (55.78)	63.48	0.63
IP-10	49.68 (21.31)	41.04	39.40 (48.58)	15.20	33.31 (43.33)	14.38	0.26
I-TAC	43.89 (17.41)	39.44	32.62 (43.47)	12.85	24.68 (31.58)	13.45	0.11
MCP-1	25.13 (16.98)	23.96	19.27 (35.27)	0.00	8.83 (13.39)	0.00	0.11
MIF	28.85 (18.26)	30.43	22.10 (35.24)	0.00	19.64 (29.91)	0.00	0.29
MIP-1 alpha	17.79 (15.52)	24.82	19.18 (27.64)	12.40	21.94 (20.64)	20.50	0.68
MIP-1Beta	20.86 (15.04)	24.05	17.27 (21.81)	16.18	23.44 (22.86)	29.23	0.57
Serpin E1	127.14 (79.14)	115.45	120.49 (50.49)	112.12	141.47 (88.28)	134.05	0.83
RANTES	253.05 (163.35)	225.42	211.29 (106.83)	176.05	197.43 (152.73)	182.11	0.87
SDF-1	43.55 (27.81)	34.14	37.65 (50.03)	17.36	30.99 (38.75)	20.43	0.34
TNF-alpha	23.31 (19.57)	22.48	22.70 (40.64)	0.00	9.77 (18.57)	0.00	0.24
sTREM-1	18.37 (17.59)	20.51	18.25 (35.58)	0.00	7.46 (13.86)	0.00	0.38

Data are presented as mean (SD) and Median., DN: Diabetic neuropathy, CF: Charcot foot disease.

**Figure 3:**
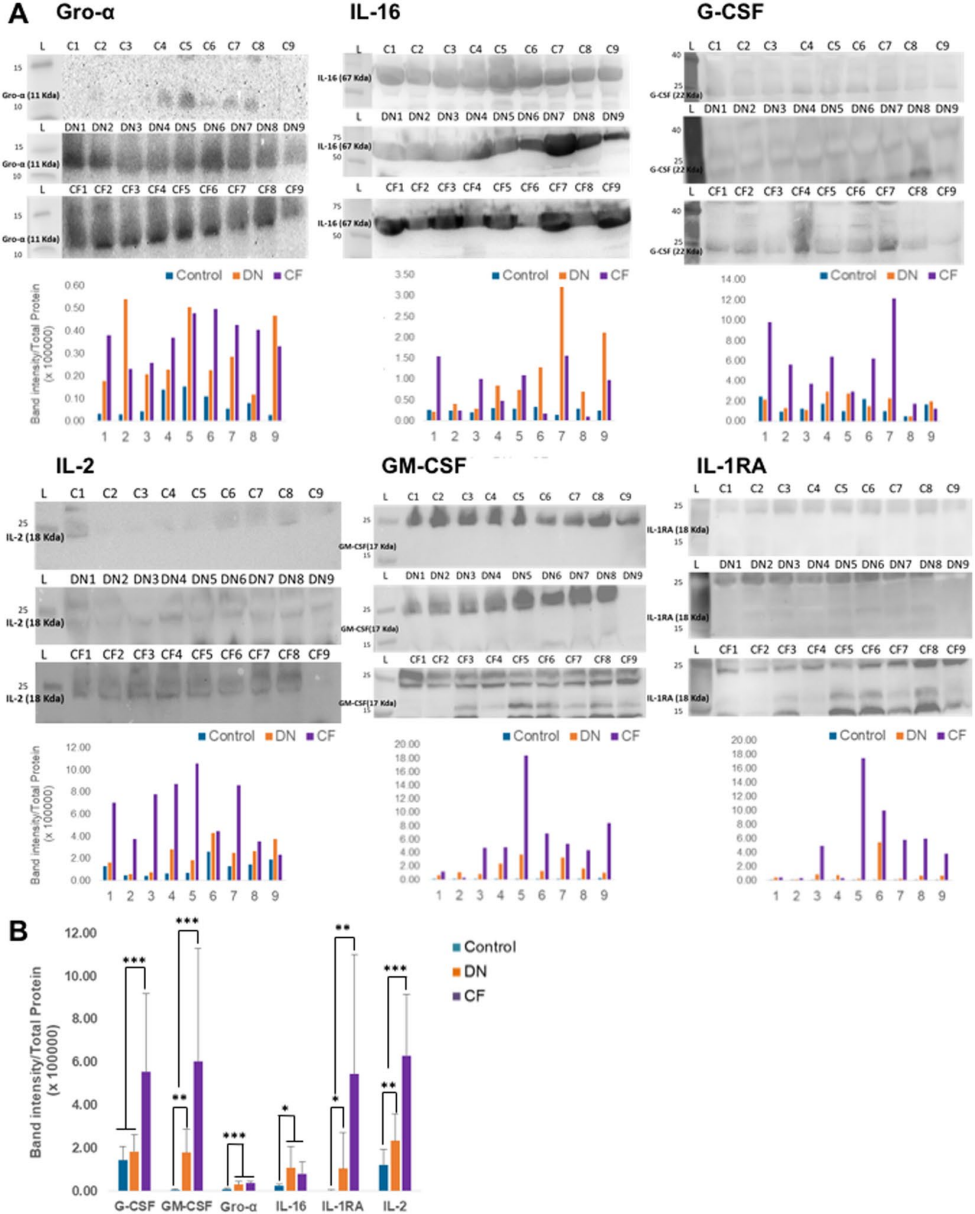
Western Blot confirmation. (**A**) Western blots against Gro-α, G-CSF, GM-CSF, IL-16, IL-2 and IL-1RA were performed. The blots were cropped to focus on the area of interest. The full blot is presented in Supplementary Figures 6–8. The size of each cytokine and the corresponding bands are highlighted on each blot at the right level on the ladder. Total protein measurement with a red Ponceau labelling has been used as loading control to normalize the band intensity of each lane. The histogram under each set of blot represent the intensity value of the band of interest normalized with the total protein. (**B**) Band intensity average per group. The band intensity average per group for each cytokine had been represented on the histogram. *p < 0.05, **p < 0.01 or ***p < 0.001.

To confirm these findings, we performed Western blotting on nine patients from each group (Fig. 3A). Total protein with red Ponceau staining was used for loading control. The band intensity has been normalized to the total protein for each lane and is displayed on the histogram on the bottom of each group of blots. There is a variability inter-patient in each different group as already display by the cytokine array. In order to be able to compare each group, we averaged the band intensity per group (Fig. 3B). G-CSF, GM-CSF, IL1-RA and IL-2 were significantly increased in the CF patients as compared to DN and controls groups. Gro-α and IL-16 were increased in T2D patients (DN and CF) as compared to healthy patients, but were no different between DN and CF groups.

### MPs effect on THP-1 human monocytic cell line and monocytes from healthy patients

**Figure 4:**
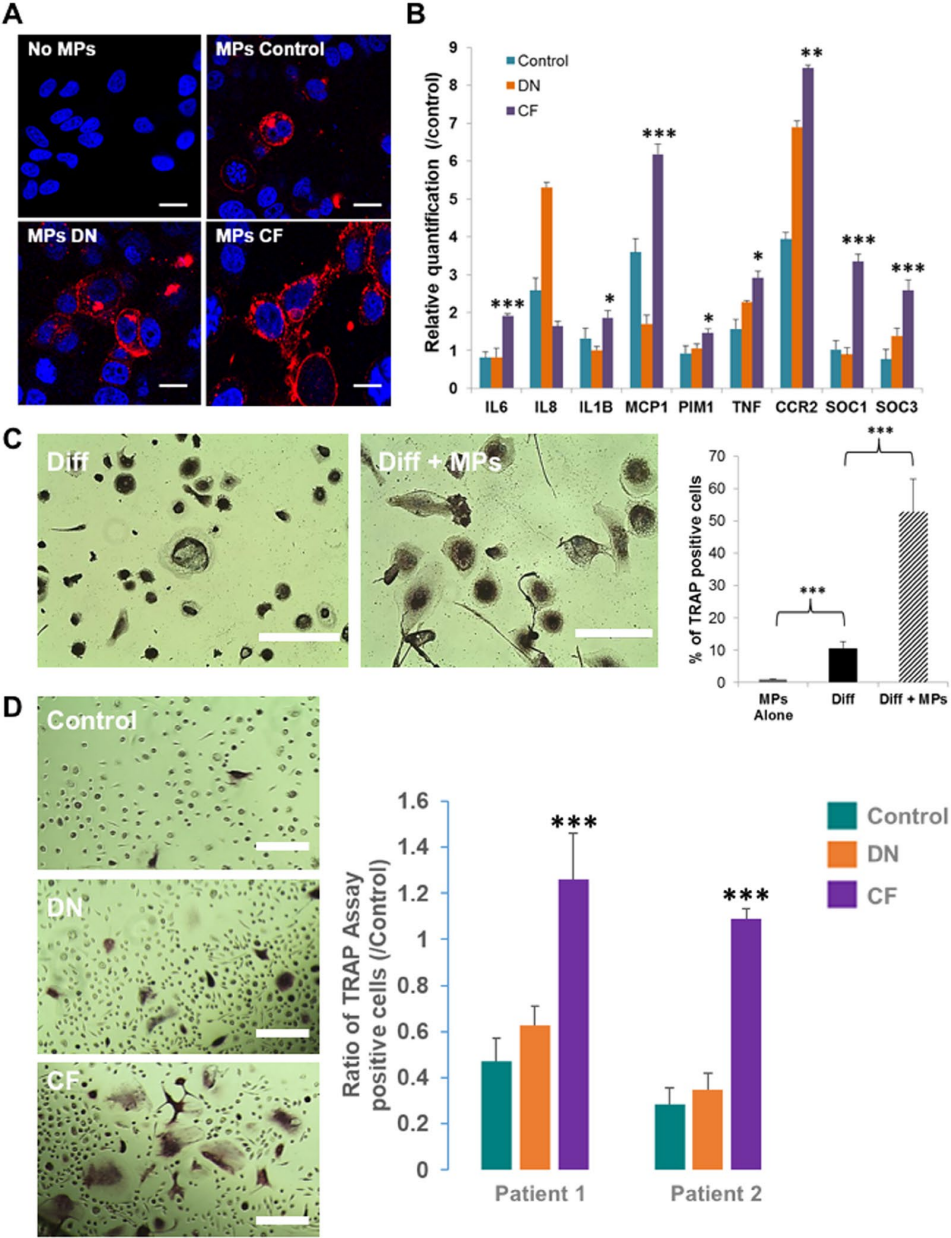
MPs effect on THP1 cell line. (**A**) Quantification of MP uptake by THP-1 cells using confocal microscopy after 3 hours of exposure. *Scale bar: 15* *µm*. (**B**) Real-time qPCR of the relative quantification of THP1 genes under control of G-CSF, GM-CSF, IL-16, IL-2 and IL-1RA pathways following 48 h of MP treatment from healthy controls (green), diabetics with neuropathy (orange) or CF (purple) patients. Relative transcript levels are represented as the ratios between the 2 subpopulations of their 2−ΔΔCp real-time PCR values. (**C**) THP1 differentiation into osteoclasts with M-CSF and sRANKL in the presence (bottom picture) or absence (top picture) of CF-derived MPs. *Scale bar: 250* *µm*. the plot represents the quantitative comparison between the percentage of TRAcP-positive cells (multinucleated osteoclast-like cells) formed in cultures with CF-derived MPs alone (grey bar) or with M-CSF and sRANKL (black bar) or with with M-CSF, sRANKL and CF-derived MPs (hatched bar). The error bar represents the difference between three independent experiments. (**D**). Monocytes differentiated into osteoclast in presence of MPs. Monocytes were sorted from two healthy patients. Osteoclast differentiation was done with M-CSF (25 ng/ml) and sRANKL (100 ng/ml) replaced every other day in presence of MPs from healthy patients (top picture), DN patient (Middle picture) or CF patient (bottom picture). Scale bar: 150 µm. The number of osteoclast was counted on 40 fields for each conditions and MPs from three patients for each group had been used. The plot represents the quantitative comparison between the percentage of TRAcP-positive cells (multinucleated osteoclast-like cells) formed in cultures with healthy- (green), DN- (orange) or CF-derived (purple) MPs compare d to the control (Ø MPs). *p < 0.05, **p < 0.01 or ***p < 0.001.

We used the human monocyte cell line THP-1 as a model to investigate the effect of MPs on a monocytic cell line. To track spontaneous uptake of MPs by THP-1, we stained MPs using WGA (wheat germ agglutinin)^19,20^. Using confocal microscopy, we visualized the uptake of MPs from the study groups (Fig. 4A). Further, we treated THP-1 cells with MPs from each of the three groups for 48 hours (6 patients per group) and examined through RT-PCR the expression of the target gene of each cytokine: IL6, IL1-β, IL-8 and MCP1 for IL-1-ra, Pim1 for GM-CSF, SOCs3 for G-CSF, SOCs1 and TNFα for IL-2 and SkP2 for IL-16 (Fig. 4B). All (except IL-8) were significantly overexpressed in THP-1 cells treated with CF-derived MPs. This experiment demonstrates that the cytokines present in the CF-derived MPs have an effect on the recipient cell.

Furthermore, we attend to evaluate the impact of the CF-derived MPs on osteoclasts production/induction. THP-1 cell line exhibits monocyte-like characteristics and has been shown to produce osteoclast precursor^24,25^. Using a counting of TRAP positive multinucleated cells, we demonstrated that MPs alone did not induce THP-1 differentiation into TRAP-positive multinucleated osteoclast-like cells (Fig. 4C). However, when THP-1 were exposed to CF-derived MPs before and during their differentiation into osteoclasts, we demonstrated an increase in the percentage of newly TRAP-positive multinucleated osteoclast-like cells (53% versus 10.52%, CF-MPs exposition *versus* no exposition, p < 0.001). MPs derived from healthy or DN patients could not reproduce the same result (Supplementary Figure 3).

To go further, we sorted monocytes from two healthy patients and induced differentiation into osteoclast in presence of MPs derived from healthy patients (control), T2D neuropathy patients (DN) or T2D with CF patients (CF). We used MPs derived from three different patients for each group on the monocytes of each patients (Fig. 4D). Only CF-derived MPs were able to increase the number of TRAP-positive multinucleated osteoclast-like cells compared to the differentiation without MPs (Ratio 1.26 ± 0.19 for patient 1 and 1.18 ± 0.04 for patient 2). Interestingly, MPs derived from healthy and DN patients are reducing the number of of TRAP-positive multinucleated osteoclast-like cells compared to the differentiation without MPs (0.47 ± 0.09 and 0.28 ± 0.07 for Control-MPs patient 1 and 2 respectively; 0.62 ± 0.08 and 0.35 ± 0.07 for DN-MPs patient 1 and 2 respectively).

## Discussion

We have demonstrated that the concentration of MPs extracted is comparable between T2D patients with DN and CF, and healthy controls. However, we showed that MPs derived from CF patients display a higher level of leukocytes- and monocytes- derived MPs. Besides, we showed that CF-derived MPs have an increased content of the pro-inflammatory cytokines: G-CSF, GM-CSF, IL-1-ra and IL-2. Moreover, when treated with CF-derived MPs, monocytes displayed increased expression of the downstream targets of these cytokines and increased differentiation into osteoclasts *in vitro*.

During the past 2 decades, there has been a significant progress in the medical management of T2D diabetes, which led to a decrease in cardiovascular-related mortality^26^. However, rare diabetes complications such as CF disease did not completely benefit from the advances in the field of diagnosis, prognosis and treatment. Circulating MPs have been proposed as new biomarkers of diabetic complications and could be potential therapeutic targets^27^. For instance, EMP levels are increased in diabetic animal models of ischemic stroke^28^, in patients with diabetic macroangiopathy^29^ and in T2D patients with coronary artery disease^30^. Platelets-derived MPs have also been shown to be involved in the progressive formation of atherosclerotic plaque and development of arterial thrombosis^31^. In the present study, we did not notice any difference in EMPs or platelet-derived MPs between diabetic patients, with DN or CF, and healthy subjects. That might be because CF disease is not a real vascular or endothelial disease but rather an inflammatory pathology that targets joints and bones. Consistently, we found an increase of MPs from leukocytes and monocytes origin in CF patients. Osteoclasts are the principal cells responsible for bone resorption in CF and are members of the monocyte and macrophage family^32^. Interestingly, non-classical monocytes population increase was reported in inflammatory and cardiovascular disease^33–35^. In our study, we found an increase in non-classical like subset population of monocytes-derived MPs (CD142^+^). To our knowledge, we are the first to report a difference of expression of these surface markers in circulating MPs. Increased levels of monocytes-MPs have been previously reported in diabetic patients with nephropathy, retinopathy or neuropathy compared to diabetic patients without microangiopathy^36^. We are the first one to report such an increase in the context of the CF disease.

The contribution of MPs in the development of CF remains unexplored. CF is a neurovascular disease; therefore, our first objective was to assess the content of CF-derived MPs in angiogenesis related proteins. Interestingly, CF-derived MPs do not seem to carry more angiogenic factor compared to MPs from healthy and DN patients. Then, we evaluated the content of inflammatory cytokines in the MPs. To our knowledge, we are the first to report that MPs exhibit a high content of inflammatory cytokines and may enhance osteoclasts differentiation in CF disease. Four cytokines -G-CSF, GM-CSF, IL-1-ra and IL-2 - were significantly increased in CF-derived MPs. Interestingly, the formation of multinucleated osteoclasts capable of bone resorption is induced by GM-CSF^37^. G-CSF also stimulates the growth of osteoclast progenitors that further differentiate into osteoclast-like cells^38^. Additionally, the short-term administration of G-CSF induces bone resorption in patients^39^. Short term G-CSF treatment also induce a decrease of osteoblasts and stimulation of osteoclasts interrupting bone remodeling balance^40^. Thus, the increased levels of G-CSF and GM-CSF in CF derived MPs could play a major role in accelerating monocyte differentiation into osteoclasts. IL-1RA is one of the receptors present on the membrane of monocytes and might play a role in osteoclast differentiation. In a recent longitudinal study of diabetic patients with CF, Folestad *et al*. reported that IL-1RA was significantly higher in CF patients at 4 months after inclusion^41^. A plausible explanation could be that circulating MPs merge with the cell membrane of monocytes once they bind to IL-1RA receptors, which induces the penetration of other cytokines into the monocytes and activation of osteoclastic differentiation pathways. IL-2 in combination with TNF- 𝛼 have a synergic effect on the positive regulation of osteoclast^42^. In osteopetrotic rats, IL-2 has been shown to correct the bone resorption^43^. IL-16 was elevated only in T2D patients (DN and CF) as compared to controls, knowing that IL-16 is known to be involved in the selective migration of CD4 T cells and participating in many inflammatory diseases^44^. *In vivo* studies are mandatory to understand the full potential of MPs to participate to the physiopathology of CF as just treating monocytes with CF-MPs seems to be a limited method. Adaptive immunity appears crucial for bone resorption and all the pro-inflammatory found up-regulated in CF-MPs have been shown to be associated with bone resorption^44^.

Previous studies have identified several systemic serological markers in acute CF. Petrova *et al*. reported that tumour necrosis factor- 𝛼 (TNF- 𝛼) and interleukin-6 are markedly elevated at the onset of the disease. Moreover, both markers correlated with C-terminal telopeptide, a marker of bone turnover^7^. Interestingly, *in vitro* inhibition of TNF- 𝛼 in monocytes of CF patients decreased bone resorption^45^. We could hypothesize that MPs might serve as serological markers in acute CF by an easy screening of G-CSF, GM-CSF, IL-1-ra, and IL-2 level in the patients circulating MPs. Besides, previous studies showed that the serum concentration of some cytokines, such as TNF-alpha and IL-6, was higher in the patients with CF. However, we were not able to demonstrate an increase in the serum level of G-CSF, GM-CSF, IL-1-ra and IL-2 in CF patients.

The critical balance between osteoblast-mediated bone formation and osteoclast-mediated bone resorption is the hallmark of many bone disease including CF. A large number of biological pathways that have not yet been clearly elucidated controls this process. The importance of the inflammatory cytokines such as TNF-α, IL-1β, and IL-6 has been demonstrated to be involved in the Charcot foot disease pathophysiology. However, the stepwise induction of them and the role of the other cytokines has not been addressed yet. Our study demonstrates that circulating MPs should also be taken into account while studying CF disease. While our study demonstrated that CF-MPs can have a role in the osteoclast differentiation *in vitro*, it seems mandatory to explore their role *in vivo*. Finally, while local inflammation is universally present, a genetic predisposition may initiate or aggravate the disease in susceptible individuals. Several osteoprotegerin (OPG) gene polymorphisms have been reported in Polish and Italian Population^46,47^. However, more recent approaches of understanding diabetes complications, such as epigenetics^48^ and omics^49^, have not been explored yet in CF disease.

## Conclusion

In conclusion, we revealed that MPs from diabetic patients with CF have a high content of inflammatory cytokines G-CSF, GM-CSF, IL-1-ra and IL-2, which further increased the differentiation of monocytes into osteoclasts *in vitro*. Replicating their role *in vivo* and understanding the molecular network induced by circulating MPs in the context of diabetic Charcot foot T2D is important since MPs might provide a potential diagnostic tool and possible therapeutic targets.

## Electronic supplementary material

Supplementary figures 1 to 5

Supplementary figure 6 to 8, supplementary tables 1 and 2
