# Supplementary material for: Circulating microparticles in acute diabetic Charcot foot exhibit a high content of inflammatory cytokines, and support monocyte-to-osteoclast cell induction
Source: Sci Rep. 2017 Nov 27;7:16450. doi: 10.1038/s41598-017-16365-7 (PMC5703953; doi:10.1038/s41598-017-16365-7)
Supplement: Supplementary file 1 — Supplementary figures 1 to 5 [file 41598_2017_16365_MOESM1_ESM.pdf]

Circulating microparticles in acute diabetic Charcot foot exhibit a high content of inflammatory cytokines, and support monocyte-to-osteoclast cell induction

Jennifer Pasquier, PhD[1,2]; Binitha Thomas, MSc [3*]; Jessica Hoarau-Véchot, MSc[3*]; Tala Odeh, MSc[3*]; Amal Robay, PhD[2,3]; Omar Chidiac, BSc[3]; Soha R. Dargham, MSc[4]; Rebal Turjoman, BSc[3]; Anna Halama, PhD[5]; Khalid Fakhro, PhD[3,6]; Robert Menzies, MSc [7]; Amin Jayyousi, MD [8]; Mahmoud Zirie, MD [8]; Jassim Al Suwaidi, MbChb [9]; Arash Rafii, MD, PhD [1,2]; Rayaz A Malik, MbChB, PhD [10,11]; Talal Talal, DMP [7] and Charbel Abi Khalil, MD, PhD [2,3,10,11] †

* These authors contributed equally to the work.

† **Corresponding author:** Charbel Abi Khalil. Weill Cornell Medicine-Qatar. PO box 24144. Doha-Qatar. Tel: +97444928484, Fax: +97444928422. E-mail: cha2022@med.cornell.edu

From the [1]Stem Cell and Microenvironment Laboratory, Weill Cornell Medicine-Qatar. Doha, Qatar, [2]Department of Genetic Medicine, Weill Cornell Medicine. New York- USA, [3] Department of Genetic Medicine, Weill Cornell Medicine-Qatar, Doha, Qatar, [4]Infectious Disease Epidemiology Group, Weill Cornell Medicine-Qatar. Doha-Qatar [5]Department of Physiology and Biophysics, Weill Cornell Medicine-Qatar. Doha – Qatar. [6]Sidra Medical and Research center. Doha-Qatar. [7] Department of Podiatry. Hamad Medical Corporation. Doha-Qatar. [8] Department of Diabetes and Endocrinology. Hamad Medical Corporation. Doha-Qatar. [9] Heart Hospital. Hamad Medical Corporation. Doha-Qatar. [10] Department of Medicine. Weill Cornell Medicine-Qatar. Doha-Qatar. [11] Department of Medicine. Weill Cornell Medicine. New York, USA.

Supplementary Data - 1

Supplementary Figure 1

A

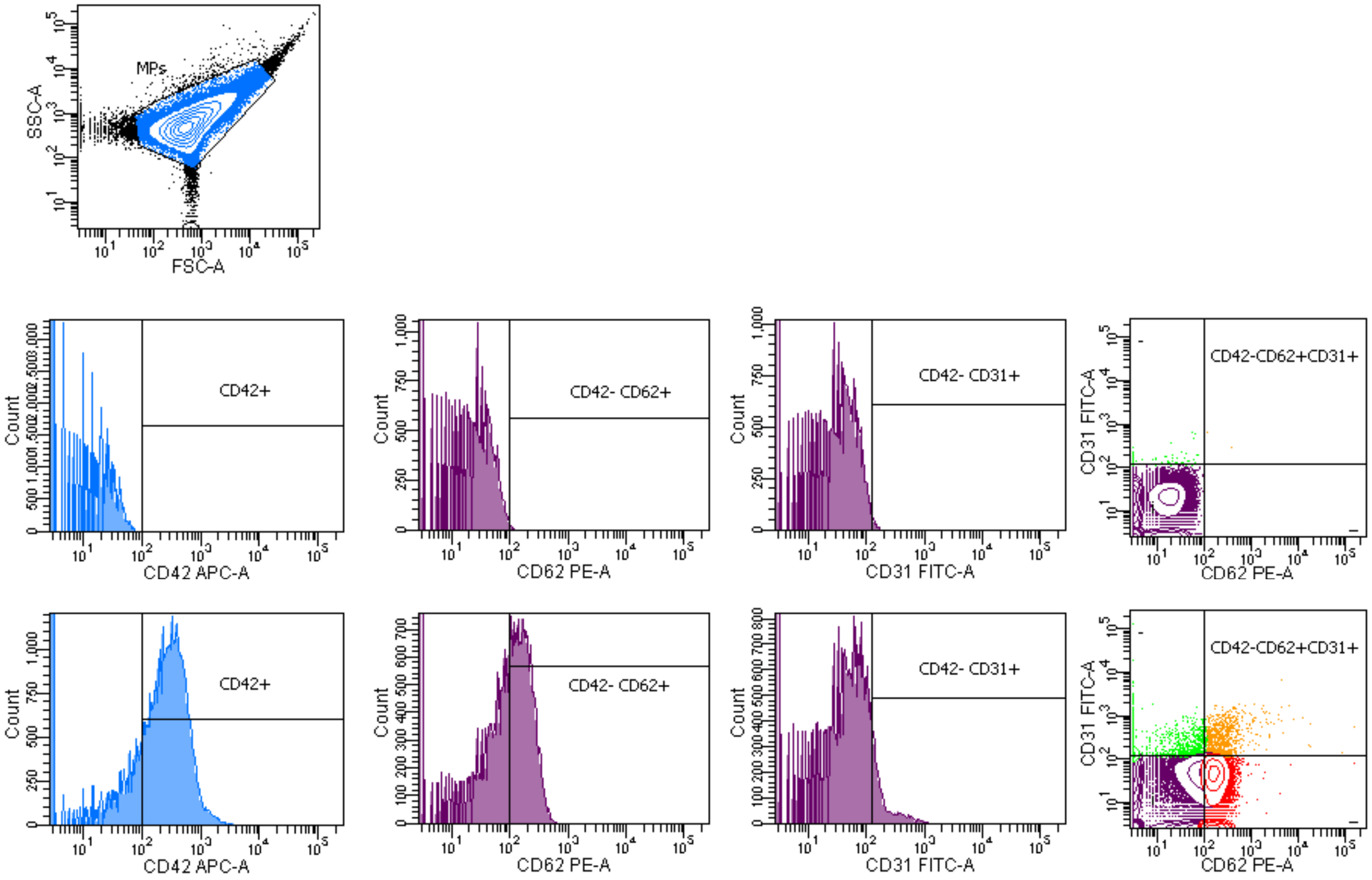

Tube: CD42/CD62/CD31

Population	#Events	%Parent	%Total
All Events	102,760	####	100.0
MPs	95,600	93.0	93.0
CD42+	40,036	41.9	39.0
NOT(CD42+)	55,564	58.1	54.1
CD42- CD62+	16,052	28.9	15.6
CD42- CD31+	3,129	5.6	3.0
-	2,065	3.7	2.0
CD42-CD62+CD31+	1,116	2.0	1.1
i	37,904	68.2	36.9
_	14,479	26.1	14.1

B

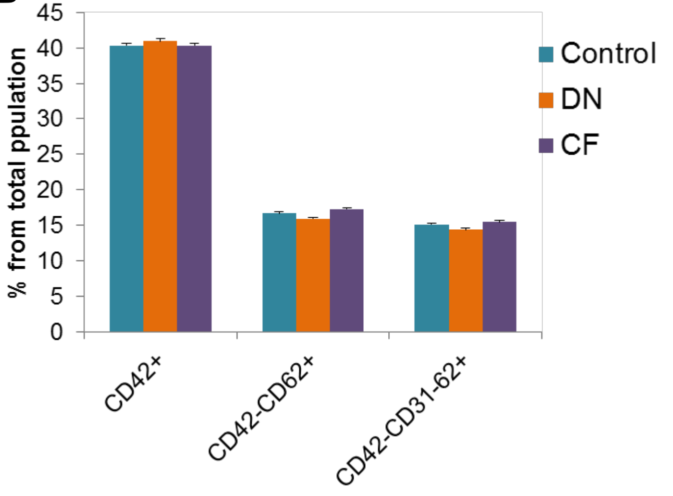

Supplementary Figure 2

A

Activin A	FGF-7/KGF	PD-ECGF
ADAMTS-1	GDNF	PDGF-AA
Angiogenin	GM-CSF	PDGF-AB/PDGF-BB
Angiopoietin-1	HB-EGF	Persephin
Angiopoietin-2	HGF	CXCL4/PF4
Angiostatin/Plasminogen	IGFBP-1	P/GF
Amphiregulin	IGFBP-2	Prolactin
Artemin	IGFBP-3	Serpin B5/Maspin
Tissue Factor/Factor III	IL-1 beta	Serpin E1/PAI-1
CXCL16	CXCL8/IL-8	Serpin F1/PEDF
DPPIV/CD26	LAP (TGF-beta 1)	TIMP-1
EGF	Leptin	TIMP-4
EG-VEGF	CCL2/MCP-1	Thrombospondin-1
Endoglin/CD105	CCL3/MIP-1 alpha	Thrombospondin-2
Endostatin/Collagen XVIII	MMP-8	uPA
Endothelin-1	MMP-9	Vasohibin
FGF acidic	NRG1-beta 1	VEGF
FGF basic	Pentraxin 3	VEGF-C
FGF-4		

B

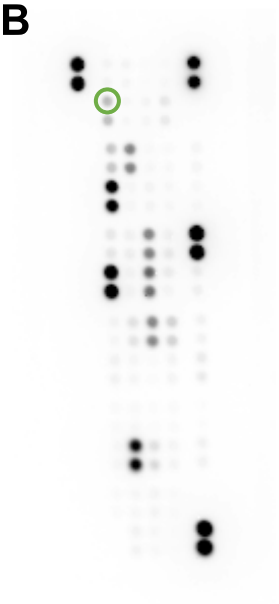

C

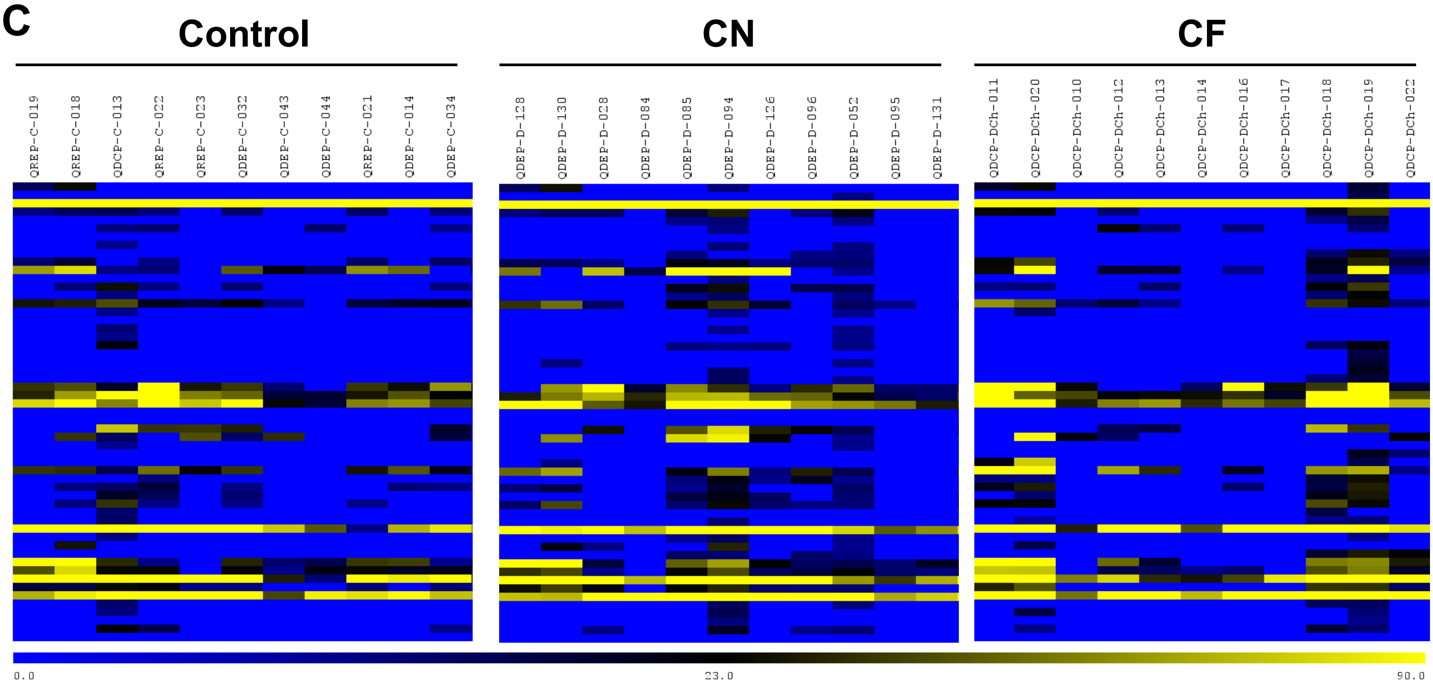

Supplementary Figure 3

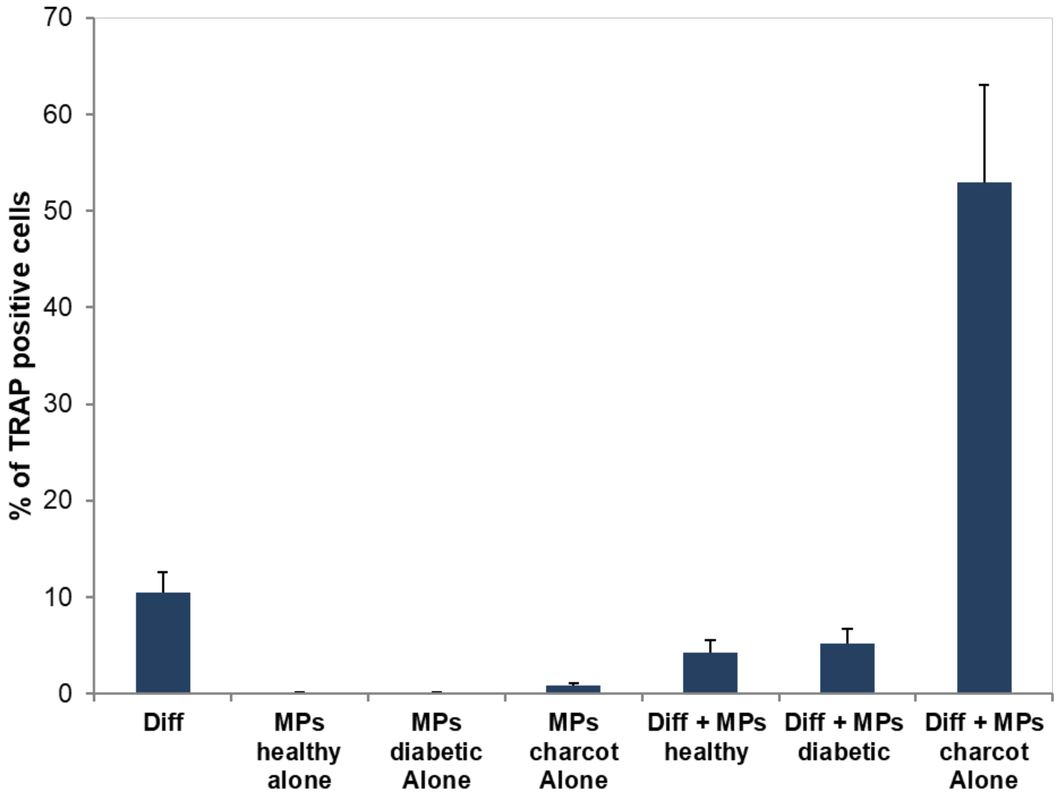

Supplementary Figure 4

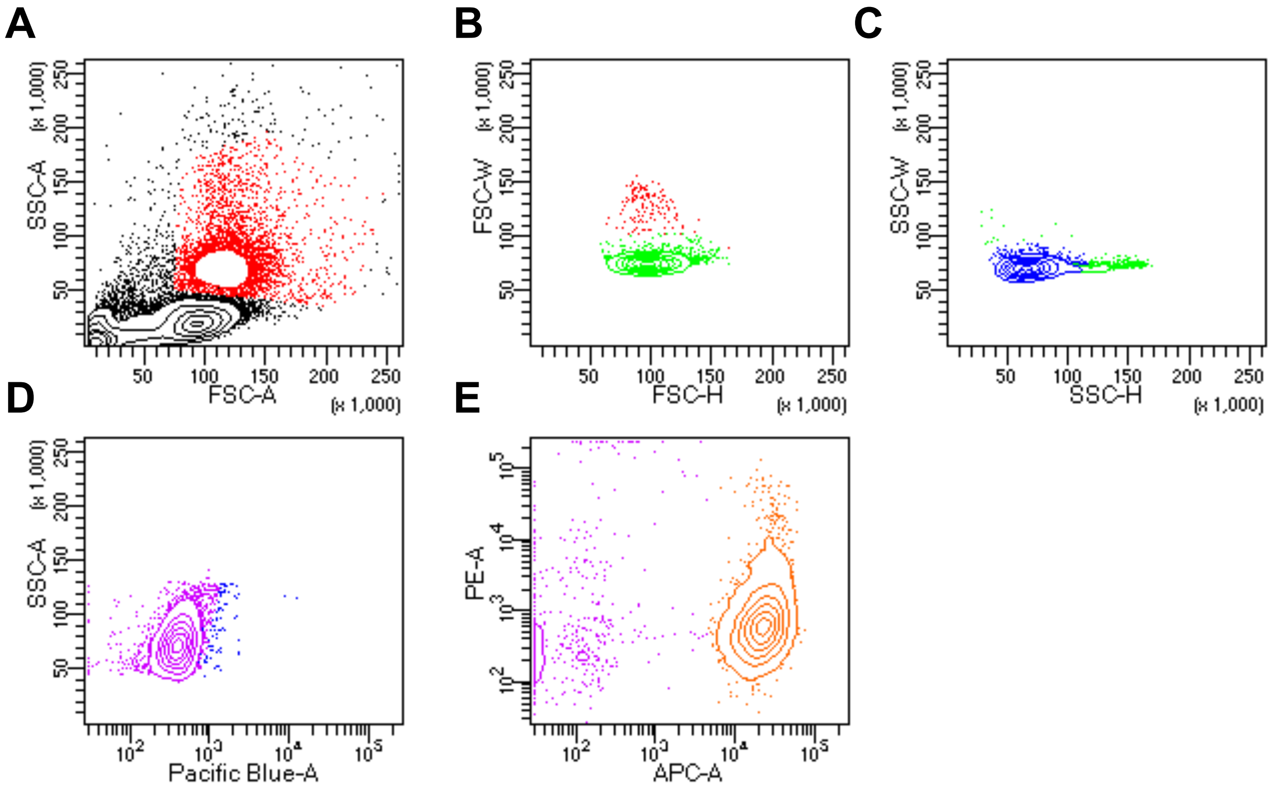

Supplementary Figure 5

Figure 1H Full Blot

Control samples

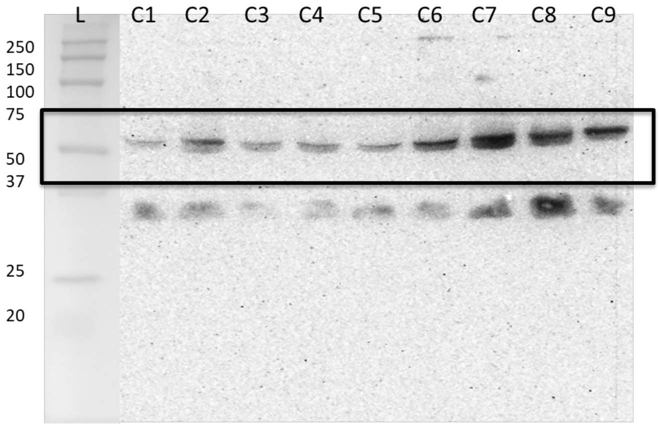

Diabetes samples

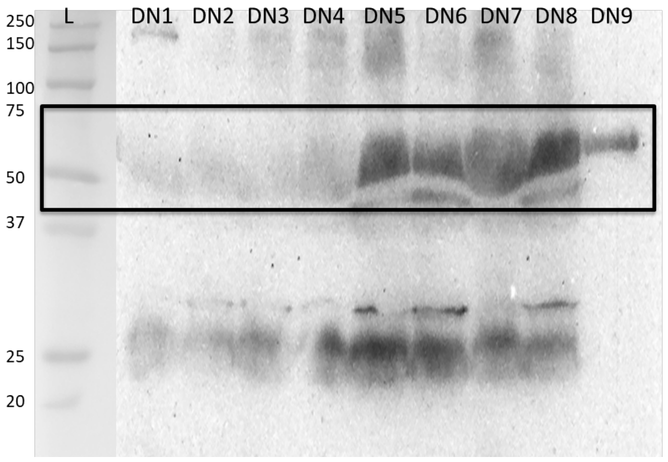

Charcot samples

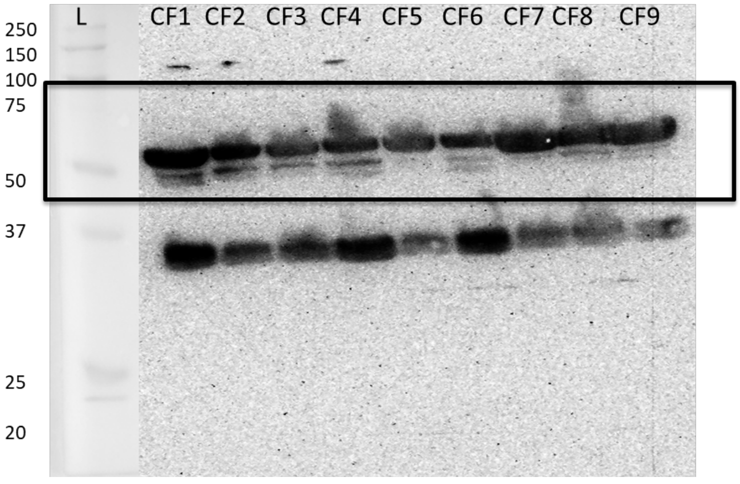

LEGENDS

- **Supplementary Figure 1. A.** Detailed flow cytometry plots of the gating protocol used to identify MPs populations. MPs from healthy controls were stained with CD42-APC, CD62-PE and CD31-FITC. Gate limits were drawn on a control isotype for each antibody. **B.** Quantifications of the MPs populations in the 3 groups, no statistically significant difference was observed.

- **Supplementary Figure 2. A.** List of cytokines tested in the angiogenesis array. **B.** The picture presents an example of a membrane. The orange circle represents the circle we draw with imageJ to measure the pixel density. The same circle is kept for the full analysis to not change the size of measurement area. **C.** Each column in the cluster graphics represents one patient and each line a cytokine. The order of cytokines is similar to the distribution in A and the intensity ranges from blue (less expressed) to yellow (more expressed).

- **Supplementary Figure 3.** THP1 differentiation into osteoclasts with M-CSF and sRANKL in the presence or absence of patients-derived MPs. Plot represents the quantitative comparison between the percentage of TRAcP-positive cells (multinucleated osteoclast-like cells) formed in cultures with MPs alone (From the 3 groups of patients, healthy, diabetic or Charcot) or with M-CSF and sRANKL (Diff) or with with M-CSF, sRANKL + patient-derived MPs (Diff + MPs). The error bar represents the difference between three independent experiments.

- **Supplementary Figure 4.** Gating strategy for monocytes sorting from patient samples. **A.** Monocytes population were gated (red population) using SSC/FSC. **B-C** Using FSC-W/FSC-h (B) and SSC-W/SSC-H (C), the doublet were excluded and only the living cells (blue population) were kept. **D.** Auto-fluorescent cells were excluded using Pacific-Blue channel. **E.** Final monocyte population was gated (orange population) as $CD14(APC)^{+}CD16(PE)^{+/-}$.

- **Supplementary Figure 5.** Full blot of the cropped blot presented in Figure 1C (**A**) and 1D (**B**). The black box represents the cropped part presented in the Figure
