# Supplementary material for: Circulating microparticles in acute diabetic Charcot foot exhibit a high content of inflammatory cytokines, and support monocyte-to-osteoclast cell induction
Source: Sci Rep. 2017 Nov 27;7:16450. doi: 10.1038/s41598-017-16365-7 (PMC5703953; doi:10.1038/s41598-017-16365-7)
Supplement: Supplementary file 2 — Supplementary figure 6 to 8, supplementary tables 1 and 2 [file 41598_2017_16365_MOESM2_ESM.pdf]

Circulating microparticles in acute diabetic Charcot foot exhibit a high content of inflammatory cytokines, and support monocyte-to-osteoclast cell induction

Jennifer Pasquier, PhD[1,2]; Binitha Thomas, MSc [3*]; Jessica Hoarau-Véchot, MSc[3*]; Tala Odeh, MSc[3*]; Amal Robay, PhD[2,3]; Omar Chidiac, BSc[3]; Soha R. Dargham, MSc[4]; Rebal Turjoman, BSc[3]; Anna Halama, PhD[5]; Khalid Fakhro, PhD[3,6]; Robert Menzies, MSc [7]; Amin Jayyousi, MD [8]; Mahmoud Zirie, MD [8]; Jassim Al Suwaidi, MbChb [9]; Arash Rafii, MD, PhD [1,2]; Rayaz A Malik, MbChB, PhD [10,11]; Talal Talal, DMP [7] and Charbel Abi Khalil, MD, PhD [2,3,10,11] †

* These authors contributed equally to the work.

† **Corresponding author:** Charbel Abi Khalil. Weill Cornell Medicine-Qatar. PO box 24144. Doha-Qatar. Tel: +97444928484, Fax: +97444928422. E-mail: cha2022@med.cornell.edu

From the [1]Stem Cell and Microenvironment Laboratory, Weill Cornell Medicine-Qatar. Doha, Qatar, [2]Department of Genetic Medicine, Weill Cornell Medicine. New York- USA, [3] Department of Genetic Medicine, Weill Cornell Medicine-Qatar, Doha, Qatar, [4]Infectious Disease Epidemiology Group, Weill Cornell Medicine-Qatar. Doha-Qatar [5]Department of Physiology and Biophysics, Weill Cornell Medicine-Qatar. Doha – Qatar. [6]Sidra Medical and Research center. Doha-Qatar. [7] Department of Podiatry. Hamad Medical Corporation. Doha-Qatar. [8] Department of Diabetes and Endocrinology. Hamad Medical Corporation. Doha-Qatar. [9] Heart Hospital. Hamad Medical Corporation. Doha-Qatar. [10] Department of Medicine. Weill Cornell Medicine-Qatar. Doha-Qatar. [11] Department of Medicine. Weill Cornell Medicine. New York, USA.

Supplementary Data - 2

Supplementary Figure 6

A – Gro-α Full Blot

Control samples

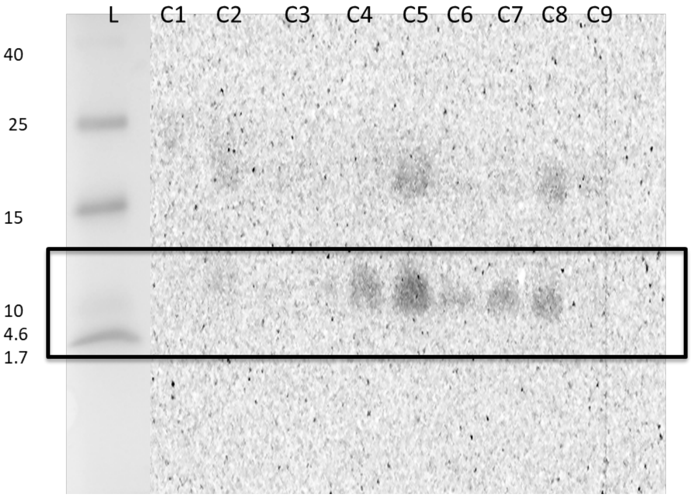

Diabetes samples

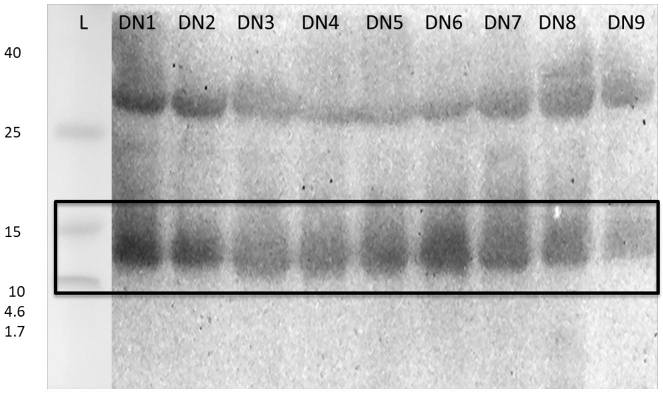

Charcot samples

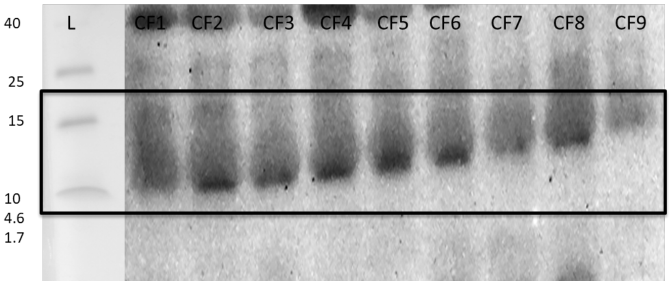

B – G-CSF Full Blot

Controls samples

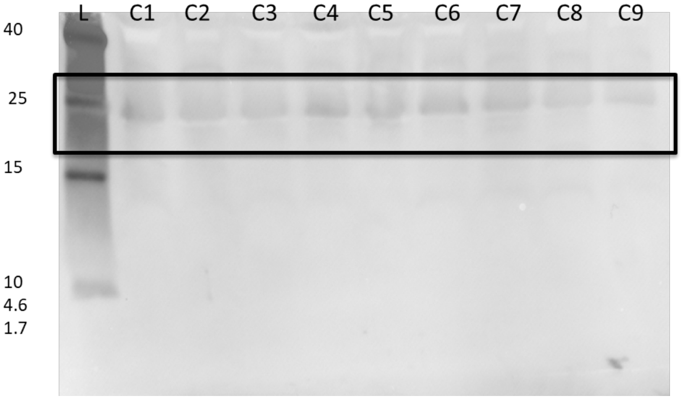

Diabetes samples

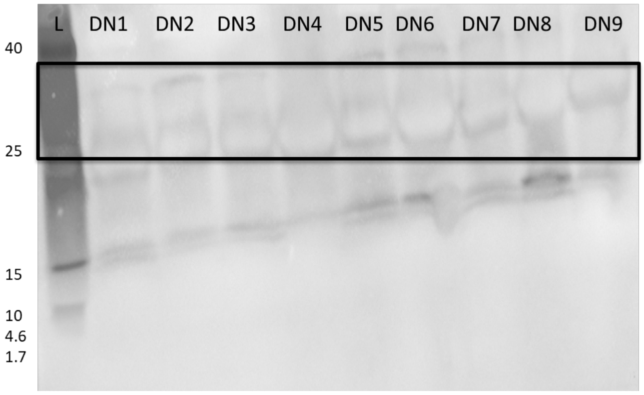

Charcot samples

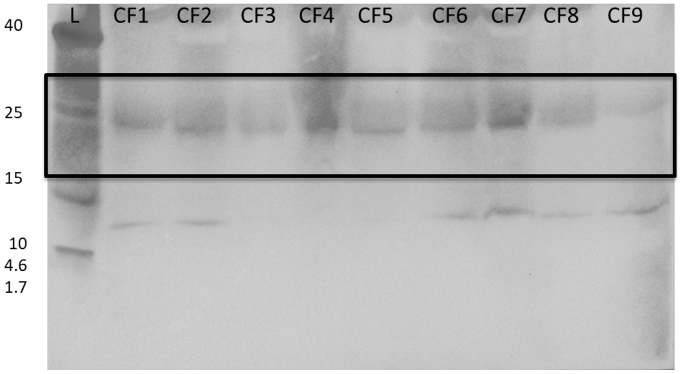

Supplementary Figure 7

A – GM-CSF Full Blot

Control samples

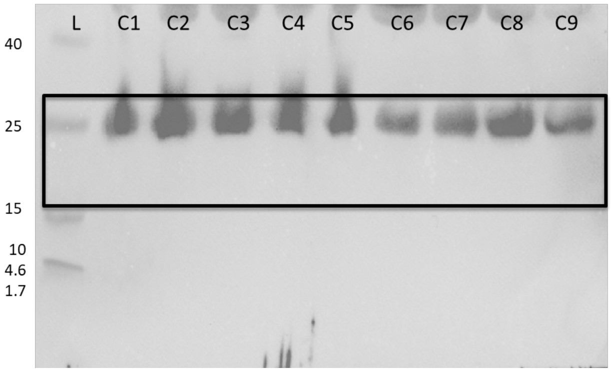

Diabetes samples

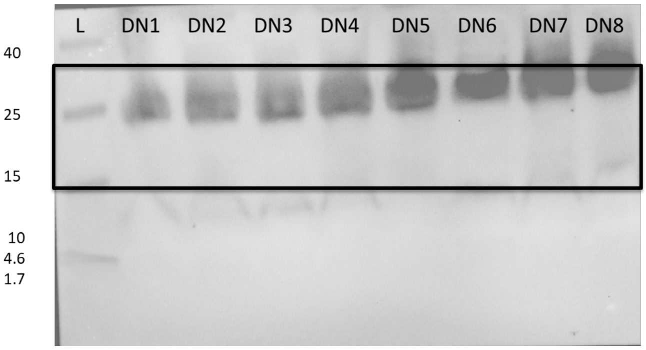

Charcot samples

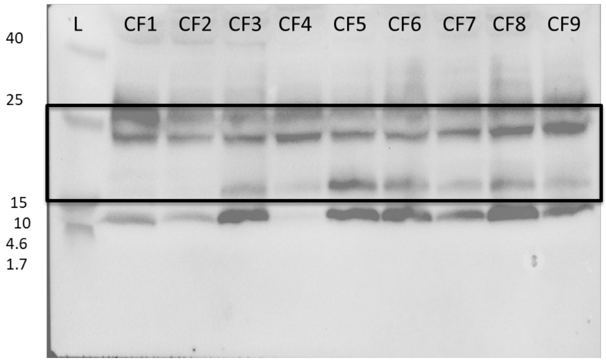

B – IL-16 Full Blot

Control samples

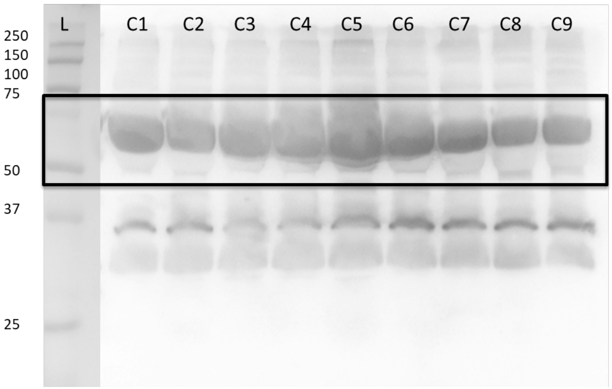

Diabetes samples

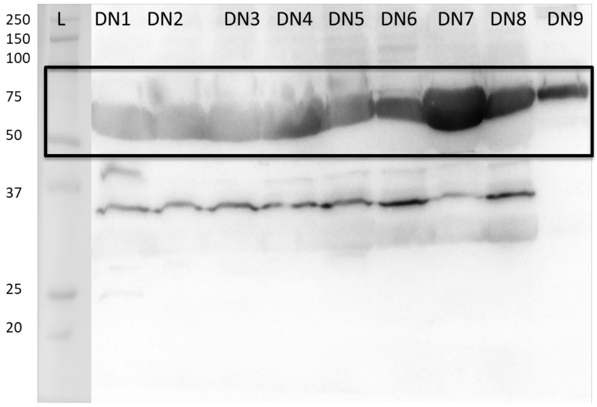

Charcot samples

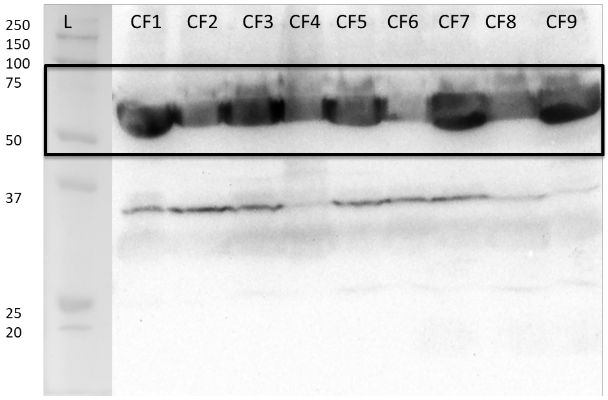

Supplementary Figure 8

A – IL-2 Full Blot

Control samples

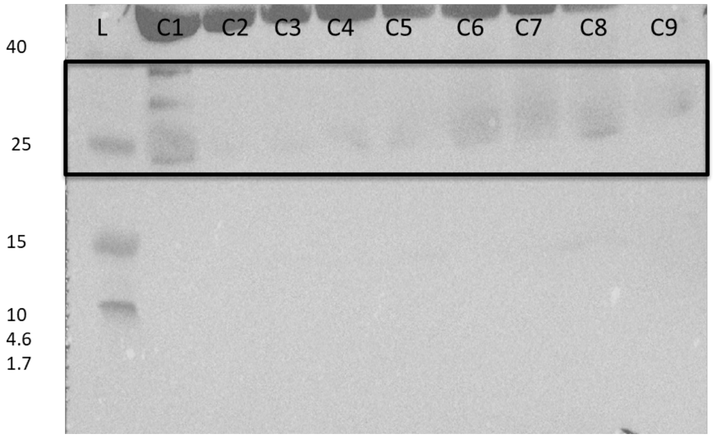

Diabetes samples

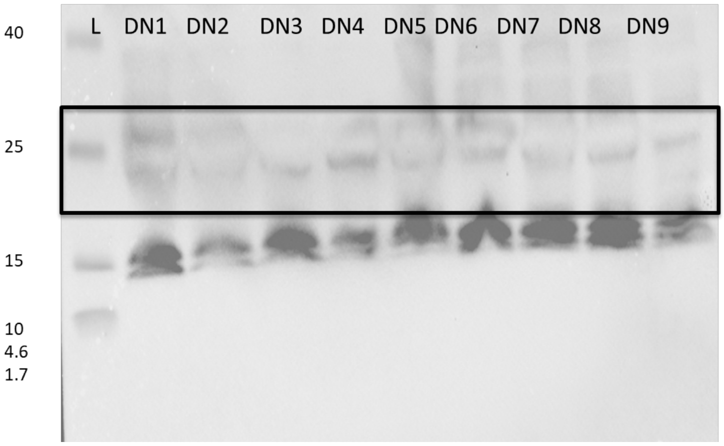

Charcot samples

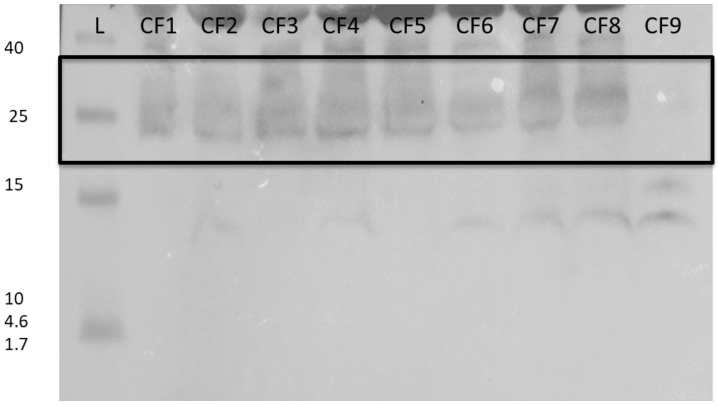

B – IL-1RA Full Blot

Control samples

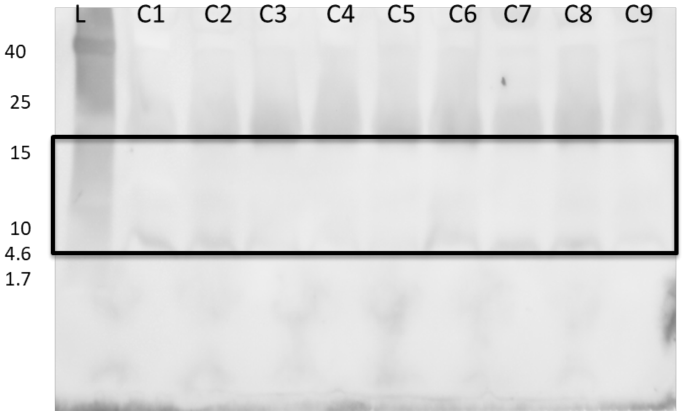

Diabetes samples

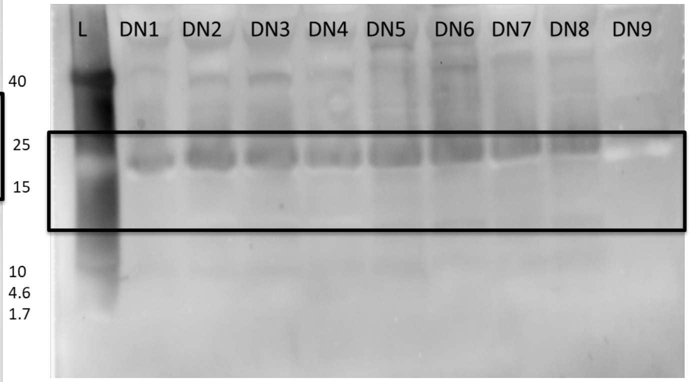

Charcot samples

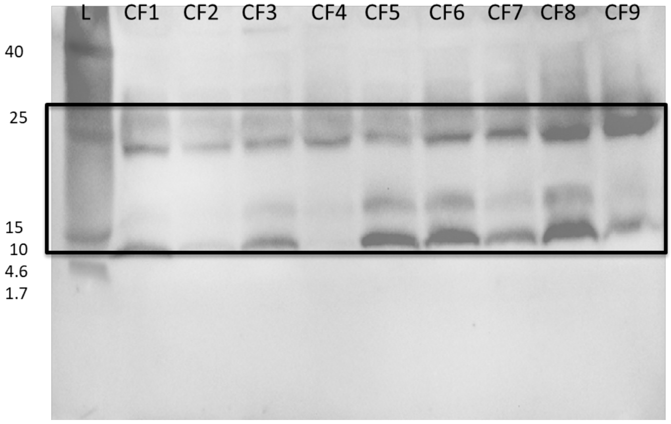

Supplementary Table 1

	Forward	Reverse
**SOCs 1**	TTTTCGCCCTTAGCGTGAAG	CATCCAGGTGAAAGCGGC
**SOCs 3**	CACCTGGACTCCTATGAGAAAGTGA	GGAGCATCATACTGATCCAGGAA
**PIM1**	AACTGGTCTTCCTTTTTGGTT	TACCATGCCAACTGTACACAC
**IL-6**	GGTACATCCTCGACGGCATCT	GT GCCTCTTTGCTGCTTTCAC
**IL-8**	ATG ACT TCC AAG CTG GCC GT	TCC TTG GCA AAA CTG CAC CT
**MCP1**	ATG AAA GTC TCT GCC GCC CT CA	GAG ATC TGT GCT GAC CCC AA
**TNF-α**	AGATGATCTGACTGCCTGGG	CTGCTGCACTTTGGAGTGAT
**CCR2**	TCACCGCTCTCGTTGGTATT	CTGAGACAAGCCACAAGCTG
**IL-1β**	AGATGATAAGCCCACTCTACAGC	CTTTAAGTGAGTAGGAGAGGTGAG
**GAPDH**	ACATCAGCCAAGTCAATGTTTCG	AGCATTAACAGCAACAATCCGG

Supplementary Table 2

	CF N=11		DN N=11		Control N=11		P-value
	Mean (SD)	Median	Mean (SD)	Median	Mean (SD)	Median	
Activin A	6.928 (11.038)	0.00	4.146 (8.584)	0.00	3.159 (7.989)	0.00	0.533
ADAMTS-1	3.322 (8.392)	0.00	0.801 (2.887)	0.00	0.000 (0.000)	0.00	0.488
Angiogenin	174.689 (46.535)	159.20	157.834 (48.218)	142.62	149.838 (36.577)	144.83	0.293
Angiopoietin-1	11.759 (14.781)	0.00	9.289 (10.118)	11.40	7.261 (7.078)	11.50	0.763
Angiopoietin-2	4.271 (8.984)	0.00	2.647 (5.040)	0.00	0.000 (0.000)	0.00	0.917
Angiostatin	7.562 (11.604)	0.00	1.712 (4.180)	0.00	3.704 (5.814)	0.00	0.108
Amphiregulin	1.044 (3.764)	0.00	0.000 (0.000)	0.00	0.000 (0.000)	0.00	0.317
Artemin	1.445 (5.212)	0.00	1.834 (4.480)	0.00	0.805 (2.903)	0.00	0.611
Coagulation Factor III	6.116 (10.588)	0.00	2.622 (4.984)	0.00	0.000 (0.000)	0.00	0.45
CXCL 16	13.602 (16.684)	0.00	9.300 (8.205)	11.42	5.351 (7.201)	0.00	0.666
DPPIV	33.629 (42.544)	18.76	36.237 (45.809)	10.56	30.315 (27.586)	22.25	0.547
EGF	4.349 (9.230)	0.00	0.942 (3.396)	0.00	0.000 (0.000)	0.00	0.27
EG-VEGF	11.614 (14.519)	11.37	6.089 (9.805)	0.00	5.694 (8.39)	0.00	0.347
Endoglin	5.052 (10.237)	0.00	2.812 (5.415)	0.00	0.864 (3.117)	0.00	0.808
Endostatin	23.491 (20.739)	17.63	18.172 (15.592)	14.53	17.978 (12.824)	19.50	0.68
Endothelin-1	3.073 (7.953)	0.00	1.616 (3.945)	0.00	0.827 (2.981)	0.00	0.87
FGF acidic	1.324 (4.772)	0.00	0.000 (0.000)	0.00	0.000 (0.000)	0.00	0.317
FGF basic	1.467 (5.288)	0.00	1.614 (3.942)	0.00	0.980 (3.534)	0.00	0.611
FGF-4	1.152 (4.153)	0.00	0.793 (2.858)	0.00	0.794 (2.864)	0.00	0.956
FGF-7	3.819 (7.505)	0.00	3.492 (5.458)	0.00	1.675 (6.041)	0.00	0.974
GDNF	3.041 (7.512)	0.00	0.000 (0.000)	0.00	0.000 (0.000)	0.00	0.149
GM-CSF	3.374 (8.359)	0.00	1.738 (4.242)	0.00	0.000 (0.000)	0.00	0.87
HB-EGF	3.905 (9.754)	0.00	1.112 (4.010)	0.00	0.000 (0.000)	0.00	0.488
HGF	4.563 (9.342)	0.00	1.887 (4.666)	0.00	0.000 (0.000)	0.00	0.526
IGFBP-1	58.249 (51.423)	38.35	35.606 (28.627)	33.83	33.350 (26.603)	29.24	0.293
IGFBP-2	58.266 (38.134)	43.28	44.048 (20.094)	46.13	45.648 (31.909)	32.45	0.522
IGFBP-3	79.587 (37.839)	68.08	67.725 (31.964)	62.27	60.915 (32.924)	58.11	0.293
IL-1β	1.656 (5.972)	0.00	0.000 (0.000)	0.00	0.000 (0.000)	0.00	0.317
IL-8	1.243 (4.480)	0.00	0.000 (0.000)	0.00	0.000 (0.000)	0.00	0.317
LAP (TGF-β1)	13.817 (22.490)	0.00	19.200 (23.460)	13.45	15.442 (23.749)	0.00	0.396
Leptin	16.050 (29.831)	0.00	20.223 (30.067)	0.00	13.878 (15.980)	13.77	0.884
MCP-1	0.971 (3.501)	0.00	0.816 (2.942)	0.00	0.789 (2.846)	0.00	0.956
MIP-1a	4.199 (8.227)	0.00	0.000 (0.000)	0.00	0.000 (0.000)	0.00	0.072
MMP-8	10.723 (21.916)	0.00	0.850 (3.063)	0.00	0.000 (0.000)	0.00	0.109
MMP-9	49.102 (44.357)	37.29	23.795 (22.673)	21.02	23.755 (17.502)	23.67	0.154
NRG1-β1	8.041 (12.401)	0.00	5.693 (8.091)	0.00	0.905 (3.263)	0.00	0.792
Pentraxin 3 (PTX3)	13.559 (14.844)	13.43	5.604 (7.634)	0.00	6.079 (7.035)	0.00	0.139
PD-ECGF	9.170 (14.118)	0.00	5.804 (8.037)	0.00	3.653 (7.077)	0.00	0.747
PDGF-AA	12.172 (17.093)	0.00	10.842 (12.212)	11.19	6.232 (10.539)	0.00	0.763
PDGF-AB/PDGF-BB	3.958 (10.343)	0.00	0.000 (0.000)	0.00	1.021 (3.681)	0.00	0.149
Persephin	4.159 (7.266)	0.00	0.964 (3.475)	0.00	1.079 (3.892)	0.00	0.168
Platelet Factor 4 (PF4)	105.107 (43.683)	104.28	96.942 (32.839)	86.88	94.003 (52.234)	84.08	0.489
PIGF	2.414 (6.262)	0.00	2.722 (5.253)	0.00	0.863 (3.110)	0.00	0.682
Prolactin	2.264 (5.569)	0.00	5.812 (10.416)	0.00	2.163 (7.800)	0.00	0.367
Serpin B5	7.501 (11.971)	0.00	3.891 (6.115)	0.00	0.000 (0.000)	0.00	0.616
Serpin E1	39.907 (41.269)	30.40	34.790 (31.977)	19.77	31.881 (29.73)	20.43	0.898
Serpin F1	30.862 (27.232)	18.53	20.592 (12.816)	16.13	23.499 (30.687)	21.42	0.521
TIMP-1	113.772 (67.329)	101.53	116.281 (52.097)	114.74	95.455 (48.833)	98.18	0.858
TIMP-4	18.193 (19.846)	13.87	13.953 (13.809)	12.90	9.753 (9.056)	11.03	0.689
Thrombospondin-1	124.479 (45.299)	116.97	109.614 (42.515)	108.74	97.789 (44.091)	85.30	0.457
Thrombospondin-2	4.285 (8.704)	0.00	1.992 (4.897)	0.00	1.010 (3.640)	0.00	0.526
uPA	4.920 (10.513)	0.00	1.211 (4.365)	0.00	0.882 (3.182)	0.00	0.27
Vasohibin	2.501 (6.510)	0.00	0.895 (3.227)	0.00	0.000 (0.000)	0.00	0.548
VEGF	5.300 (8.932)	0.00	4.201 (6.89)	0.00	3.913 (7.839)	0.00	0.851
VEGF-C	1.035 (3.730)	0.00	0.000 (0.000)	0.00	0.000 (0.000)	0.00	0.317

LEGENDS

- **Supplementary Figure 6.** Full blot of the cropped blot presented in Figure 3. (**A**) Gro-α (**B**) G-CSF . The black box represents the cropped part presented in the Figure

- **Supplementary Figure 7.** Full blot of the cropped blot presented in Figure 3. (**A**) GM-CSF (**B**) IL-16. The black box represents the cropped part presented in the Figure

- **Supplementary Figure 8.** Full blot of the cropped blot presented in Figure 3. (**A**) IL-2 (**B**) IL-1RA. The black box represents the cropped part presented in the Figure

- **Supplementary Table 1.** Primers list.

- **Supplementary Table 2. Angiogenesis array between the studied groups.** Data are presented as mean (SD) and Median., DN: Diabetic neuropathy, CF: Charcot foot disease.
